# Profiling Plasma Extracellular Vesicle Metabotypes and miRNAs: An Unobserved Clue for Predicting Relapse in Patients with Early-Stage NSCLC

**DOI:** 10.3390/cancers16223729

**Published:** 2024-11-05

**Authors:** Vivi Bafiti, Eleni Thanou, Sotiris Ouzounis, Athanasios Kotsakis, Vasilis Georgoulias, Evi Lianidou, Theodora Katsila, Athina Markou

**Affiliations:** 1 Institute of Chemical Biology, National Hellenic Research Foundation, 11635 Athens, Greece; pmpafiti@eie.gr (V.B.); souzounis@eie.gr (S.O.); 2 Lab of Analytical Chemistry, Department of Chemistry, National and Kapodistrian University of Athens, 15771 Athens, Greece; elenathanou@chem.uoa.gr (E.T.); lianidou@chem.uoa.gr (E.L.); 3 Department of Medical Oncology, University General Hospital of Larissa, 41334 Larissa, Greece; thankotsakis@hotmail.com; 4 First Department of Medical Oncology, Metropolitan General Hospital of Athens, 15562 Cholargos, Greece; georgulv@otenet.gr

**Keywords:** extracellular vesicles, miRNAs, NSCLC, metabotypes, multi-omics, liquid biopsy

## Abstract

Background and Objective: Lung cancer, the second most prevalent cancer globally, poses significant challenges in early detection and prognostic assessment. Despite advancements in targeted therapies and immunotherapy, the timely identification of relapse remains elusive. Blood-based liquid biopsy biomarkers, including circulating tumor cells (CTCs), cell-free DNA (cfDNA), circulating tumor DNA (ctDNA), circulating-free RNAs (cfRNAs), and extracellular vesicles (EVs)/exosomes, offer promise for non-invasive monitoring. Methods: We employ a comprehensive approach integrating miRNA/lncRNA/metabolomic datasets, following a mixed-methods content analysis, to identify candidate biomarkers in NSCLC. NSCLC-associated miRNA/gene/lncRNA associations were linked to in silico-derived molecular pathways. Results: For data validation, mass spectrometry-based untargeted metabolomics of plasma EVs highlighted miRNA/lncRNA/metabotypes, linking “glycerophospholipid metabolism” to *lncRNA H19* and “alanine, aspartate and glutamate metabolism” to *miR-29a-3p*. Prognostic significance was established for *miR-29a-3p*, showing lower expression in NSCLC patients with disease progression compared to stable disease (*p* = 0.004). Kaplan–Meier survival analysis indicated that patients with *miR-29a-3p* under-expression had significantly shorter overall survival (OS) (*p* = 0.038). Despite the expression of *lncRNA H19* in plasma EVs being undetected, its expression in plasma cfRNAs correlated significantly with disease progression (*p* = 0.035). Conclusions: Herein, we showcase the potential of plasma EV-derived *miR-29a-3p* as a prognostic biomarker and underscore the intricate interplay of miRNAs, lncRNAs, and metabolites in NSCLC biology. Our findings offer new insights and avenues for further exploration, contributing to the ongoing quest for effective biomarkers in early-stage NSCLC.

## Simple Summary

Prognosis, early detection, and relapse identification are jointly crucial factors for effective lung cancer treatment. Blood-based liquid biopsy, a non-invasive method, can provide solutions to the deadlocks in the management of this widespread cancer type by eliciting biomarkers. In this research, an integrated approach is developed, which combines the use of databases and experimental validation to find adequate biomarkers in non-small-cell lung cancer. The arising results, concerning miR-29a-3p in exosomes and lncRNA H19 in cfRNA, widen the horizons for identifying and exploiting promising biomarkers in non-small-cell lung cancer.

## 1. Introduction

Lung cancer is the second most frequently diagnosed type of cancer. The relative 5-year survival rate increases from 6% for distant-stage disease to 33% for regional-stage disease and 60% for local-stage disease [1]. While lung cancer therapies have made great strides with the discovery of various targeted therapies [2] and the effective use of immunotherapy in some patient groups, currently used disease monitoring methods and treatment regimens lack the ability to detect relapse early [3]. Lung cancer is divided into two main types, which are non-small-cell lung cancer (NSCLC) and small-cell lung cancer (SCLC). The three main types of NSCLC are adenocarcinoma, squamous cell carcinoma, and large-cell carcinoma. NSCLC is only detected when the disease is already advanced [4].

Blood-based liquid biopsy biomarkers such as circulating tumor cells (CTCs), circulating tumor DNA (ctDNA), circulating cell-free RNAs (cfRNAs), and extracellular vesicles (EVs)/exosomes are potential indicators of tumor burden in cancer patients [5,6]. These minimally invasive “liquid biopsies” have attracted considerable attention due to their obvious clinical significance for personalized medicine, such as the identification and stratification of cancer patients [7]. The use of liquid biopsies for the early detection of lung cancer is of great public interest [8] but faces major challenges in terms of the standardization of pre-analytical conditions and methodologies and the diagnostic specificity and sensitivity of biomarkers.

EVs carry various types of cargo molecules including RNAs, lipids, DNAs, proteins, and metabolites and due to the protection of these cargo molecules by their lipid bilayer membrane, EVs have attracted considerable attention as a component of liquid biopsies [9,10]. Tumor cell-derived EVs have previously been shown to contain disease-related markers [6], and circulating EVs derived from tumor cells could be a new minimally invasive diagnostic tool for identifying asymptomatic cancer patients [10,11,12,13]. To date, there have been few studies on the systematic screening of EVs associated with prognosis and response to the treatment of lung cancer [14,15,16,17].

Metabolomics analysis of EVs has gained interest in cancer research [18,19], as metabolomics studies allow for the simultaneous analysis of thousands of different endogenous metabolites in a given biological sample. In terms of responses to treatment, several groups have investigated metabolomic changes in cancer cells [20,21,22,23], suggesting that an unbiased metabolomic investigation of cancer cell-derived EVs is essential to identify novel cancer biomarkers for prognosis, prediction, and therapeutic responses in several cancers, including lung cancer [24,25].

In addition to metabolites, microRNAs (miRNAs) are also important components of EVs and are more stable in circulation than cell-free miRNAs [26]. EVs are enriched with noncoding RNAs, including miRNAs. Extracellular miRNA mainly exists in the EVs, so they are thought to be selectively classified as EVs [27]. Both miRNA and pre-miRNA can be secreted into exosomes and microvesicles in protein-bound and protein-free forms. In addition to being packed into exosomes or microvesicles, extracellular miRNAs can be loaded into high-density lipoprotein (HDL) [28] or bound by AGO2 protein outside of vesicles [29]. Recent studies have shown that exo-miRNAs can be used as diagnostic and prognostic biomarkers in human malignant tumors, including breast and lung cancer [30,31,32,33]. Moreover, in lung cancer, EV miRNA expression levels have been associated with resistance to targeted therapy [34,35,36].

Herein, we designed, employed, and optimized a strategy coupling a mixed-methods content analysis (i.e., gold standard approach for content analysis) to a metabotype approach in non-small-cell lung cancer (NSCLC) for bias minimization in the selection of EV miRNAs as predictors of relapse in patients with early-stage NSCLC. For this, data and text mining led to candidate miRNAs and lncRNAs, further supported by in silico-derived molecular pathways. The latter were validated by mass spectrometry-based untargeted metabolomics in plasma EVs. Next, the informative relationships through which metabotypes (individual metabolomic profiles) are connected to miRNAs/lncRNAs were interrogated to reveal miR-29a-3p, which was differentially expressed and detected in early-stage NSCLC plasma EVs vs. healthy individuals. This holistic strategy presents a great opportunity to unveil patterns and provide new insights for NSCLC biology.

## 2. Materials and Methods

### 2.1. Clinical Samples

Thirty-two patients with early-stage NSCLC were enrolled in the study. From these patients, *n* = 32 peripheral blood samples (25 mL in EDTA tubes) were prospectively collected at baseline (pre-surgery), and the peripheral blood samples from *n* = 10 healthy donors (HDs) were used as controls. All patients gave written informed consent to participate in the study, which was approved by the Ethics and Scientific Committee of the Metropolitan General Hospital of Athens. All HDs had no known illness or fever at the time of the blood draw, no history of malignant disease, were ≥35 years old, and 52.6% were female and 47.4% were male. The main patient characteristics of the clinical samples are summarized in Table 1.

### 2.2. Isolation of Extracellular Vesicles

EV isolation was previously described [37] and performed herein for the most efficient RNA isolation using the Macherey-Nagel™ Exosome Precipitation Solution for Serum/Plasma (Düren, Nordrhein-Westfalen, Germany). This polymer-based method was chosen due to its high recovery rate and ability to preserve the integrity and biological relevance of EVs. Size exclusion chromatography further confirmed EV isolation in addition to liquid chromatography with tandem mass spectrometry (Supplementary Materials). Considering that transmission electron microscopy (TEM) suffers from drawbacks such as time-consuming processes, low analysis throughputs, and potential imaging artifacts, high-sensitivity nano-flow cytometry analysis was applied to more thoroughly reveal the concentration and size distribution of EVs (NanoFCM Inc., Nottingham, United Kingdom). EVs were also characterized by automated digital holographic microscopy (Agilent BioTek Lionheart FX, Agilent, Santa Clara, CA, USA) with DiO staining for lipid bilayer detection (MISEV2023). For this, an automated image segmentation and quantification framework based on a multitask learning (MTL) convolutional neural network was employed. The MTL model was built on a modified U-Net architecture [38] incorporating transfer learning, residual connections, and a regression block for the quantification of EVs. For network training, EVs from cell culture supernatants were isolated and stained with DiO for fluorescence imaging at 20× magnification (*n* = 115). Images were augmented (10 per sample), producing 102,465 sub-images resized to 128 × 128 pixels. Image preprocessing involved green channel enhancement with CLAHE [34] and denoising. Ground truth masks were generated using an automated pipeline based on pixel intensity distributions. The architecture featured a Vgg16 pre-trained encoder [39], a connection block, and a U-Net-based decoder. Residual connections were incorporated, and a regression block was added for counting. The MTL model performed two tasks, namely image segmentation to produce masks via the U-Net decoder and EV counting via the regression head. Overall performance was evaluated using the Dice coefficient [40,41] and the intersection over union (IoU) [42] for segmentation. Outputs were compared to BioTek Gen5 (Agilent, Santa Clara, CA, USA), EVAnalyzer [43], and NanoFCM Software V1.17 (NanoFCM Inc., Nottingham, United Kingdom) for quantification against an external test of human plasma samples. Hyperparameter tuning was performed for model prediction optimization. The optimal MTL model selected along with the tuned parameters was employed for EV quantification (NSCLC patient plasma sample images). The models were built in R language v4.1 utilizing TensorFlow [44] and Keras libraries (https://keras.io, accessed on 1 November 2022). For statistics, the Wilcoxon test was performed in RStudio Server Version 1.4.1717, “Juliet Rose” (df86b69e, 24 May 2021) for CentOS 8, R version 4.1.0 (18 May 2021)—“Camp Pontanezen”.

### 2.3. Isolation of Total RNA and cDNA Synthesis

Total RNA isolation from EVs was carried out utilizing the RNeasy Mini Kit (Qiagen, Hilden, Germany) following the protocols provided by the manufacturer. Subsequently, cDNA was synthesized from total RNA using the Engineered M-MLV Reverse Transcriptase Basic Kit (EnzyQuest, Crete, Greece) in a reaction with a total volume of 20 μL according to the manufacturer’s instructions, whereas in the case of miRNAs, the cDNA was synthesized using the High-Capacity cDNA Reverse Transcription Kit (Applied Biosystems™, Massachusetts, MA, USA) and miRNA-specific stem-loop primers in a reaction with a total volume of 17 μL. For recovery estimation, 0.2 nM of an exogenous synthetic miRNA, Caenorhabditis elegans miR-39 (*cel-miR-39*), was added to each sample as an external control, as previously described [45,46].

### 2.4. Quantification of miRNA Expression by RT-qPCR

The expression levels of *miR-29a-3p*, *miR-191*, and *cel-miR-39* were quantified by RT-qPCR using TaqMan microRNA assays (Applied Biosystems, Waltham, MA, USA) according to the manufacturer’s protocols. RT-qPCR was performed in a final volume of 10 μL containing 1 μL of cDNA template, 5 μL of Platinum™ Quantitative PCR SuperMix-UDG (Invitrogen, Waltham, Massachusetts, USA), 0.5 μL miRNA-specific primer, and 3.5 μL (DEPC)-treated H_2_O. All reactions were performed using the Cobas^®^ 4800 (Roche Diagnostics, Rotkreuz, Switzerland). The reaction mixture was incubated at 95 °C for 10 min, followed by 45 cycles of 95 °C for 15 s and 60 °C for 1 min. Expression values were normalized to miR-191, which was found to be a suitable reference miRNA. The expression levels of *miR-29a-3p* were normalized using the 2^−ΔΔCt^ approach to the expression of miR-191.

The expression levels of lncRNA *H19* and *B*_2_*M* were quantified by RT-qPCR. RT-qPCR was performed in a final volume of 10 μL with 1 μL cDNA template and 9 μL mix. All reactions were performed using the Mic Real qPCR Cycler (Bio Molecular Systems, Upper Coomera QLD, Australia). The reaction mixture was incubated at 95 °C for 10 min, followed by 45 cycles of 95 °C for 15 s and 60 °C for 1 min. The expression levels of *H19* were normalized using the 2^−ΔΔCt^ approach to the expression of *Β*_2_*Μ*.

### 2.5. Data and Text Mining

We applied a mixed-methods content analysis, a gold standard approach for a content analysis consisting of deductive (quantitative) and inductive (qualitative) phases, taking into account contemporary definitions. For data and text mining, as well as data analysis, the peer-reviewed literature, omics datasets, and clinical trial outcomes were mined to investigate miRNAs/lncRNAs/plasma metabotypes of NSCLC. We have also developed a novel framework to meet our analytical demands by exploring data (both context and content). The literature data from Scopus and PubMed/MEDLINE were queried. Scopus and PubMed/MEDLINE are the largest citation and abstract databases of the peer-reviewed literature. To account for selection biases, private and publicly available texts have been assessed (based on the inclusion/exclusion criteria set, as well as the keywords and MeSH terms in question; www.nlm.nih.gov/mesh, accessed on 8 October 2022; Supplementary Table S1). We questioned the interim output further for open data (yes/no), sample size (validated by a power analysis), research approach, and publication impact/metrics. Studies that failed to meet inclusion criteria or studies on non-human samples were excluded. Two co-authors (V.B. and T.K.) co-analyzed the interim and final outputs, and then the percentage of inter-rater agreement was calculated. To account for biases, Cohen’s kappa statistic and percentage agreement were also determined with multi-categorical ratings. Candidate miRNAs/lncRNAs/plasma metabotypes were identified.

For gene expression analysis, an external cohort of NSCLC patients was employed, matching the histological subtypes of our cohort and also serving as the only publicly available dataset of the largest sample size; the GDD53627 (GSE10245) dataset from the Gene Expression Omnibus (GEO) [47] repository that consists of adenocarcinoma (*n* = 40) and squamous cell carcinoma (*n* = 18) data.

Differential gene expression (DGE) analysis was performed for the two subtypes within the GEO platform through the Geo2R tool (http://www.ncbi.nlm.nih.gov/geo/geo2r/, accessed on 28 September 2022) using the cut-off values of *p*-adj. < 0.05 and fold change > |2|. Genes were noted either as up- or down-regulated. Next, the miRTarBase [48] was mined for gene–miRNA pairs, taking into account only the experimentally validated human gene–miRNA pairs, resulting in *n* = 93 miRNAs being not only NSCLC-related but also associated with the NSCLC subtypes in question. To determine miRNA/lncRNA associations in NSCLC, the LncTarD 2.0 platform [49] was used. We retrieved all the experimentally supported functional lncRNA target datasets (key targets and biological functions driven by disease-related lncRNAs and lncRNA-mediated regulatory mechanisms in human diseases). Then, NCSLC filters were applied to ensure that DEG/miRNA/lncRNA associations also provided underlying information regarding NSCLC subtypes (adenocarcinomas and squamous cell carcinomas).

lncRNA and miRNA datasets were cross-linked, validated, and enriched by pathway analysis and text mining, then filtered by statistical significance. miRNAs were interrogated further using miRPathDB [50], listing in silico-derived molecular pathways. A pathway-centric approach was followed, selecting only those pathways with strong evidence of association through experimental validation. Hence, we identified the pathways in which our miRNAs were expected to be over-represented based on a statistical significance filter with an adjusted *p*-value < 0.05.

**Figure 1:**
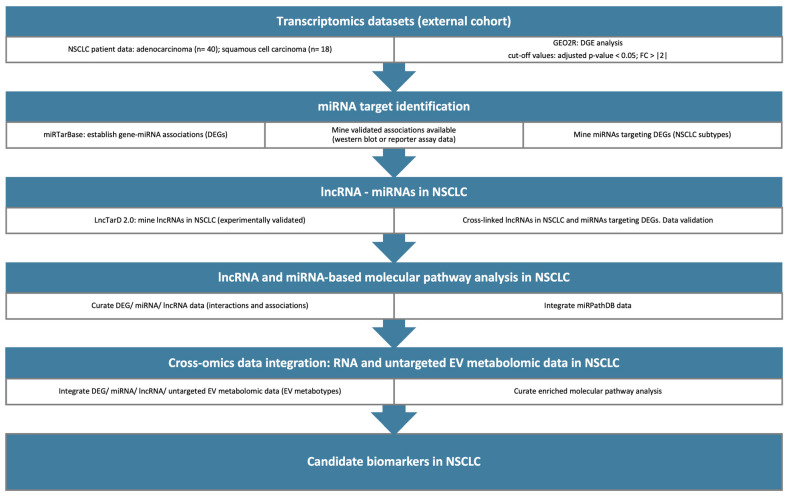
A graphical representation of the in silico pipeline designed and employed to identify miRNAs and lncRNAs that can serve as candidate biomarkers for NSCLC. Transcriptomics datasets were retrieved from the GEO database and the DGE analysis was performed with the GEO2R tool. NSCLC lncRNAs were mined through LncTarD2.0 and then cross-linked with miRNAs-DEGs. Pathway analysis was conducted based on the identified lncRNAs and miRNAs. Next, cross-omics data integration between RNA and EV metabotypes was implemented to reveal candidate biomarkers in NSCLC.

Untargeted mass spectrometry-based metabolomics was performed on plasma EVs to a. provide an external validation method for the computationally identified miRNA-related pathways enabling pathway-based integration and b. assess the discrimination ability of our cohort based on the histological subtypes of NSCLC patients. Given that histological subtypes serve as known independent prognostic factors, metabotypes were obtained and tested to see if they could stratify NSCLC patients based on their prognosis (typically, survival rates are higher for patients with squamous cell carcinomas when compared to patients with adenocarcinomas). Metabolite Set Enrichment Analysis (MSEA) was also performed to identify biologically meaningful patterns significantly enriched in our dataset. miRNAs/lncRNAs/metabolites were linked through their enriched molecular pathways, data, and text mining, then filtered by statistics. Our pipeline for the identification of miRNAs and lncRNAs as candidate biomarkers is summarized in Figure 1 and provided in detail in the Supplementary Materials and Supplementary File 1.

### 2.6. Untargeted Metabolomics

For quenching and extracting the EV metabolites, we added ice-cold 80:20 mass spectrometry-grade methanol/water (*v*/*v*) to each sample prior to snap freezing in liquid nitrogen for 1 min (three repeats). Samples were then thawed on ice, followed by vortex mixing and sonication between each cycle. Samples were centrifuged at −5 °C at 15,000× *g* for 10 min and supernatants were loaded into Amicon Ultra 3 kDa tubes (Merck Millipore, MA, USA) as per the manufacturer’s instructions. Flow-throughs were collected and dried down (lyophilized) in a centrifugal vacuum evaporator for 18 h. No heating was applied during the drying process. Next, samples were reconstituted and filtered through a 0.22 μm filter and fortified with stable isotope-labeled standards before injection. Quality control and internal standard samples were prepared as described [51,52]. The analysis was carried out using a Thermo Scientific Vanquish LC coupled to Orbitrap Exploris 240 MS (Thermo Fisher Scientific, Waltham, MA, USA). An electrospray ionization interface was used as an ionization source. Analysis was performed in positive and negative ionization modes under polarity switching. The UPLC was performed using a slightly modified version of the protocol described by Catalin et al. (UPLC/MS Monitoring of Water-Soluble Vitamin Bs in Cell Culture Media in Minutes, Water Application note 2011, 720004042en). Peak areas were extracted using Compound Discoverer 3.3 (Thermo Scientific). Data were processed using Compound Discoverer 3.3 (ThermoFisher Scientific) and Skyline 22.2. The identification of compounds was performed at four levels; level 1: identification by retention times (compared against in-house authentic standards), accurate mass (with an accepted deviation of 3 ppm), and MS/MS spectra; level 2a: identification by retention times (compared against in-house authentic standards), accurate mass (with an accepted deviation of 3 ppm); level 2b: identification by accurate mass (with an accepted deviation of 3 ppm) and MS/MS spectra; and level 3: identification by accurate mass alone (with an accepted deviation of 3 ppm) (Supplementary Files 2 and 3, as well as deposited data in the EMBL-EBI MetaboLights database with the identifier MTBLS11489 [53]).

### 2.7. Statistical Analysis

Statistical analysis was performed using the SPSS (version 26) statistical package. For the statistical analysis, we divided NSCLC patients into two different groups, high-expression and low-expression groups, using the median ΔΔCq of noncancerous samples for each miRNA studied at the corresponding cut-offs. Our data were evaluated related to the expression of *miR-29a-3p* by normalizing to the expression of *miR-191* and using the 2^−ΔΔCt^ method, as described in detail by Livak and Schmittgen [54]. Similarly, we analyzed all our data related to the overexpression of lncRNA H19 by normalizing to the expression of *B_2_M* and using the 2^−ΔΔCt^ method. The Mann–Whitney test was used to analyze the difference in miRNA expression levels between the groups and the comparison between the different baseline characteristics.

For metabolomics, test groups were cross-compared first to gain insights into the NSCLC metabotypes [52]. Univariate and multivariate statistical analysis were applied where appropriate, followed by Bonferroni correction. A critical significance threshold was set at <0.05, including FDR correction. For comparative analysis, log2-fold change calculations were performed, along with principal component analysis (PCA) and ortho partial least squares discriminant (OPLS-DA) analysis (Metaboanalyst 5.0) [55]. Only metabolites with annotation levels 1 and 2a were selected for subsequent enrichment analysis (Supplementary Files 2 and 3). Metabolite Set Enrichment Analysis (MSEA) was performed using Metaboanalyst 6.0 [56] employing pathway-associated metabolite sets (SMPDB). Data were normalized to the sample median, followed by log10 transformation and Pareto scaling. For the interrogation of metabolic pathways, the mummichog algorithm was applied, enabling one-step functional analysis through tandem mass spectra feature tables [57]. The top 10 most significantly associated *m*/*z* features were input into the mummichog algorithm v.2. The KEGG (Kyoto Encyclopedia of Genes and Genomes) database was selected as the pathway library of interest. Only those metabolic pathways containing at least 3 significant metabolites were considered. The significance threshold was set at a *p*-value < 0.05, including FDR correction.

## 3. Results

### 3.1. Gene–miRNA–lncRNA Associations Unveil Candidate NSCLC Biomarkers

**Figure 2:**
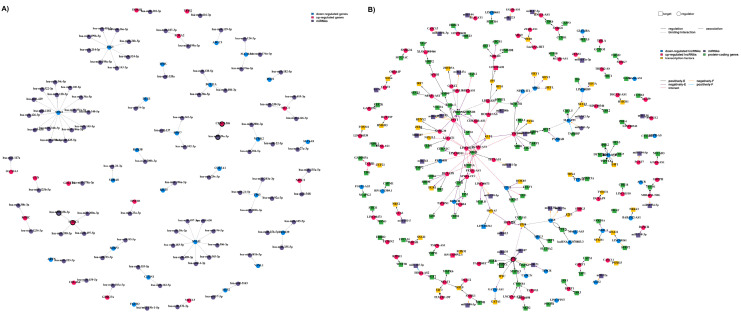
Network representation of the NSCLC-related gene, miRNA, and lncRNA associations. (**A**) miRNA–gene associations (a directed graph); red nodes: up-regulated genes; blue nodes: down-regulated genes; purple nodes: miRNAs. The nodes are interconnected with arrowed edges indicating the direction of the association. (**B**) miRNA/gene/lncRNA associations; two types of nodes are depicted, also in different shapes. Rectangular nodes: target elements; circular nodes: regulatory elements; blue nodes: down-regulated lncRNAs; red notes: up-regulated lncRNAs; yellow nodes: transcription factors; purple nodes: miRNAs; green nodes: protein-coding genes. Three edge types indicate the relationships among the nodes in question; solid edges: regulatory relationships; dotted edges: binding or interaction; double-dashed edges: associations; edge colors: regulation direction; black edges: an increase in expression (positively E); purple edges: a decrease in expression (negatively E); orange edges: a decrease in function (negatively F); blue edges: a positive function (positively F); and red edges: an interaction between nodes (interact). Nodes with bold outlines: candidate biomarkers.

Differential analysis of gene expression in adenocarcinoma and squamous cell carcinoma NSCLC patients has uncovered a set of *n* = 228 differentially expressed genes (DEGs), comprising *n* = 196 down-regulated and *n* = 112 up-regulated genes. Our findings, illustrated in the volcano plot (Supplementary Figure S1), suggest distinct regulatory patterns. miRTarBase analysis identified *n* = 93 miRNAs targeting *n* = 42 genes, as depicted in Figure 2 (DEG/miRNA associations in a directed graph). Network A showcases gene–miRNA associations, highlighting that among *n* = 42 DEGs, *n* = 23 were up-regulated, whereas *n* = 70 were down-regulated in NSCLC adenocarcinoma compared to squamous cell carcinoma. Of note, three central hubs—*JAG1*, *SNAI2*, and *SOX2*—emerged in network A, all being down-regulated. LncTarD 2.0 retrieved NSCLC-associated lncRNAs (rectangle nodes). The resulting two networks yielded a curated list of associations among miRNAs/genes/lncRNAs associations (Supplementary Table S2).

**Figure 3:**
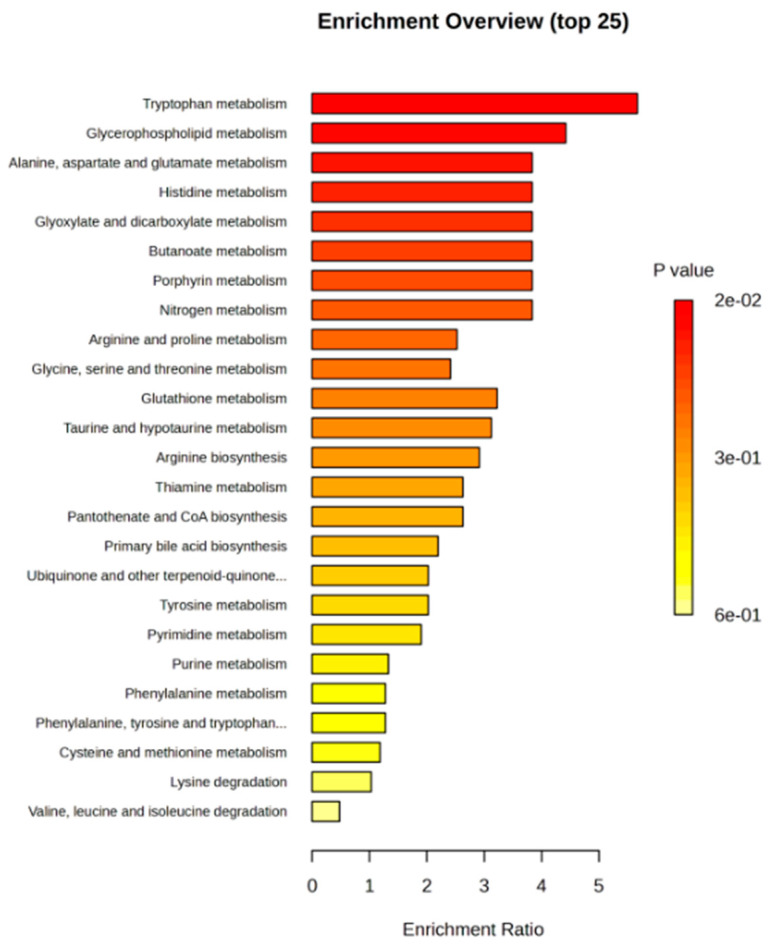
Untargeted metabolomics in plasma EVs from NSCLC patients reveals key perturbed metabolic pathways. Metabolite Set Enrichment Analysis (MSEA) was performed using Metaboanalyst v.6. The enriched pathways were ranked by significance, as indicated by the color scale (top ten pathways with *p*-values < 0.05).

To validate in silico-derived molecular pathways, untargeted mass spectrometry-based metabolomics in plasma EV-cross-linked metabotypes with miRNA/lncRNA-enriched pathways were generated by our computational approach. First, EVs were quantified per sample, and then disease progression or survival was considered (Supplementary Figure S2). Overall, two pathways were revealed to be enriched both in the metabolomics datasets of our cohort and the miRNAs/lncRNAs linked to the external NSCLC cohort, namely “alanine, aspartate and glutamate metabolism” and “glycerophospholipid metabolism” pathways (Figure 3 and Supplementary Files 3–5). *miR-29a-3p* was found to be over-represented in the “alanine, aspartate and glutamate metabolism” pathway, whereas *miR-338-3p* was over-represented in the “glycerophospholipid metabolism” pathway (Supplementary Table S3). The latter was linked to *MACC1* and hence, to *lncRNA H19*. Candidate biomarkers have bold outlines in both networks A and B.

### 3.2. Prognostic Significance of Differentially Expressed miR-29a-3p in Plasma EVs of Early-Stage NSCLC Patients

A positive or negative association was explored for *miR-29a-3p* and *miR-191* in plasma EVs. Over-expression and under-expression were estimated by evaluating the differences in *miR-29a-3p* expression levels between plasma EVs of NSCLC patients before surgery (*n* = 31) and HDs (*n* = 10). The expression levels of *miR-29a-3p* in plasma EVs of NSCLC patients were significantly different from HDs (*p* = 0.004, Supplementary Figure S3).

**Figure 4:**
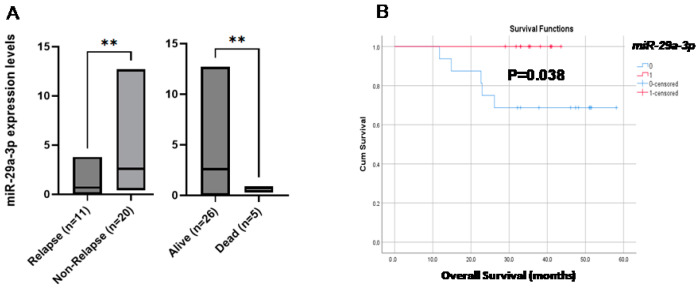
(**A**) Relative-fold change (2^−ΔΔCq^) of *miR-29a-3p* in EVs from early-stage NSCLC patient samples in terms of relapse and survival, (**B**) Kaplan–Meier estimates of OS for NSCLC patients with respect to *miR-29a-3p* expression. ** *p* ≤ 0.01.

The correlation of *miR-29a-3p* expression levels with the patients’ clinical outcome revealed that patients who had a progression of the disease had significantly lower expression levels than patients who had stable disease (*p* = 0.002, Figure 4A). Kaplan–Meier survival analysis and log-rank tests were performed by using patients’ postoperative survival. Kaplan–Meier survival curves demonstrated that patients with *miR-29a-3p* under-expression had significantly shorter overall survival (OS) than those with *miR-29a-3p* over-expression (*p* 0.038, Figure 4B), whereas there was no correlation with disease-free intervals (DFIs).

### 3.3. Expression of LncRNA H19 in NSCLC Plasma EVs and Plasma cfRNA

RT-qPCR was used to detect the expression of *lncRNA H19* in EVs from NSCLC patients (*n* = 31) and HDs (*n* = 10) (Supplementary Figure S4). The results showed that the expression of *lncRNAH19* was not detected in any of the plasma EV samples. We further evaluated the expression of *lncRNA H19* in plasma cfRNAs of the same patients, and we observed that there is a statistically significant difference in expression between patients that had disease progression than patients who had stable disease (*p* = 0.035). However, the expression levels of *lncRNA H19* in plasma samples were not correlated with DFIs and OS.

## 4. Discussion

EVs are an essential component of carcinogenesis and are found in cancer-releasing mediators that affect tumor progression through their ability to transfer their cargo between cells [58]. To date, most studies in EVs have examined nucleic acids or proteins in EVs [59], but metabolomics analysis in EVs provides in-depth network analysis by integrating DNA–RNA–protein–metabolite interactions.

In the present study, comprehensive EV metabolomic profiling was performed to identify and internally validate candidate biomarkers for the early detection of relapse in NSCLC patients and their interactions with ncRNAs. Our study shows that metabolomics in plasma EVs (untargeted analysis) links “glycerophospholipid metabolism” to *lncRNA H19* and “alanine, aspartate and glutamate metabolism” to *miR-29a-3p*. Both glycerophospholipid and alanine, aspartate, and glutamate metabolism have been showcased in lung cancer, mainly in serum samples [60,61]. Hence, our findings highlight a critical aspect of NSCLC’s metabolic reprogramming. Tryptophan metabolism has been identified as the pathway with the most significant enrichment (with a *p*-value of 0.015). This result is consistent with previous studies on NSCLC patient serum samples, which suggest that alterations in tryptophan metabolism play a critical role in tumor progression, underscoring the diagnostic and prognostic potential of L-tryptophan [62,63]. However, our analysis did not reveal any significant connection between tryptophan metabolism and miRNAs/ ncRNAs.

Glycerophospholipid metabolism, pivotal for membrane biosynthesis and cellular signaling, has been previously implicated in cancer; herein, we extend this association into the realm of EVs, suggesting that alterations in this pathway could be mediated or regulated by *lncRNA H19*. Given that *lncRNA H19* has been implicated in various oncogenic processes, including proliferation, apoptosis resistance, and metastasis, its interaction with glycerophospholipid metabolism may provide novel insights [64]. For *miR-29a-3p* and the metabolism of alanine, aspartate, and glutamate, it is known that those amino acids play vital roles in cancer metabolism, including supporting the tricarboxylic acid (TCA) cycle and nucleotide synthesis, which are essential for the high proliferative demands of cancer cells. The association of *miR-29a-3p* with these metabolic pathways reinforces its potential as a biomarker, given its known role in modulating gene expression involved in cancer progression and its demonstrated presence in lung cancer [65,66,67]. The interplay between *miR-29a-3p* and these metabolic pathways further suggests a nuanced mechanism where miRNA regulation could influence the metabolic rewiring characteristic of NSCLC.

To our knowledge, this is the first time that *miR-29a-3p* has been studied in plasma EVs from early-stage NSCLC patients. Few studies have reported its role in lung cancer in cell lines [66,67,68,69]. According to our results, the expression levels of *miR-29a-3p* in plasma EVs from NSCLC patients were significantly different from HDs (*p* = 0.004), and patients with advanced disease had significantly lower expression levels than patients with stable disease (*p* = 0.002). This finding is consistent with other studies using plasma or tissue samples, reporting that *miR-29a-3p* was abnormally low in a number of human malignancies, including papillary thyroid carcinoma, hepatocellular carcinoma, gastric cancer, and breast cancer [70].

According to our results, the expression of *lncRNA H19* in plasma samples from patients with NSCLC was prognostically insignificant, but there was a statistically significant difference between NSCLC patients in whom the disease was advanced and patients in whom the disease was stable. Studies have shown that highly expressed H19 in plasma could be a potential biomarker for the diagnosis of breast cancer and lung cancer [71]. Luo et al. have shown that based on the relative expression levels of plasma H19, significantly higher levels were observed in the NSCLC group than in the benign disease group [72].

Circulating exosomal *lncRNA H19* has been described as a potential biomarker with diagnostic and prognostic value in gastric cancer (GC) [73] and breast cancer [74]. However, an interesting finding in the current study was that the expression of *lncRNA H19* was not detected in the corresponding plasma EVs samples from NSCLC patients. This observation could be due to the nature of the samples, as serum samples were used in the previous studies, and the early stage of the disease is another reason for the limited amount of EVs in the bloodstream.

Further controlled studies with a larger number of patients are needed to confirm these observations. Our study combines metabolomics with miRNAs and EVs in early-stage NSCLC. It is expected that this combination will be of great benefit and contribute to the optimal use of treatment and management strategies for patients with an increased risk of metastasis.

## 5. Conclusions

Taking everything into consideration, the present study combines metabolomics with miRNAs and EVs in early-stage NSCLC. The results, related to miR-29a-3p and lncRNA H19, highlight their potential as promising biomarkers, which could play a key role in the treatment and management of patients with an increased risk of metastasis.

## Figures and Tables

**Table 1:** Characteristics of clinical samples.

Patient	Age (years)	Smoking	Tumor Size (cm)	Histological Type	TNM	Disease Stage	Relapse	Death	DFS	OS
1	60	-	2.6	SCC	T1aN0M0	IA	1	0	14.2	58.1
2	54	yes	1.8	SCc	T1bN0M0	IA	0	0	51.6	51.6
3	47	-	7	AdenoCA	T2bN0M0	IB	0	0	51.4	51.4
4	74	-	2	SCC	T1N0M0	IA	1	0	15.0	51.2
5	67	-	4	AdenoCA	T2aN0M0	IB	1	1	13.8	15.8
6	52	yes	8	SCC	T4N1M0	IIIA	1	1	10.0	14.8
7	76	yes	3.8	SCC	T2aN2M0	IIIA	1	1	6.5	11.7
8	68	yes	4	SCC	T2aN0M0	IB	1	1	14.0	26.1
9	73	-	1.8	AdenoCA	T1bN1M0	IIB	1	1	31.5	40.4
10	65	-	9	SCC	T4N1M0	IIIA	0	0	48.2	48.2
11	39	no	3	AdenoCA	T1cN0M0	IA	1	0	35.4	47.5
12	61	yes	3.5	SCC	T2aN0M0	IB	0	0	47.4	47.4
13	73	yes	5.5	SCC	T3N0M0	IIB	1	0	47.2	47.2
14	66	yes	4	LCNEC	T3N1M0	IIIA	1	1	8.7	22.9
15	75	yes	3	AdenoCA	T2aN2MO	IIIA	1	1	12.1	36.1
16	73	no	5.5	SCC	T3N0M0	IIB	1	0	12.4	46.1
17	59	no	4	AdenoCA	T2N1M0	IIB	1	1	12.2	21.8
18	48	-	1.2	AdenoCA	T1bN0M0	IA	0	0	41.2	41.2
19	67	yes	1	AdenoCA	T1aN0M0	IA	0	0	40.9	40.9
20	73	-	7.4	SCC	T4N0M0	IIIA	0	1	31.8	31.8
21	70	-	2.8	SCC	T1cN0M0	IA	0	0	40.8	40.8
22	64	-	3.4	AdenoCA	T2aN1M0	IIB	1	1	11.4	29.0
23	57	yes	2.1	AdenoCA	T1cN1MO	IIB	1	0	38.2	38.2
24	69	yes	3.4	SCC	T2aN2M0	IIIA	1	1	9.5	22.6
25	63	yes	3.2	AdenoCA	T2aN0M0	IB	0	0	37.8	37.8
26	72	yes	2.8	SCC	T1cN0M0	IA	1	0	7.3	37.8
27	62	-	3.5	AdenoCA	T2aN0M0	IB	0	0	35.5	35.5
28	72	-	2.5	AdenoCA	T1cN0M0	IA	0	0	35.2	35.2
29	75	yes	2.5	SCC	T1cN0M0	IA	0	0	35.2	35.2
30	79	yes	2.1	AdenoCA	T2aN0M0	IB	0	0	33.0	33.0
31	56	yes	3.1	AdenoCA	T2aN0M1		0	0	33.0	33.0
32	73	no	8.5	SCC	T4N0M0	IIIA	0	0	33.0	33.0

TNM: tumor–nodes–metastasis classification system; DFS: disease-free survival; OS: overall survival.

## Data Availability

The data presented in this study are available upon request from the corresponding author.

## Supplementary Materials

The following supporting information can be downloaded at: https://www.mdpi.com/article/10.3390/cancers16223729/s1. Supplementary Table S1: MeSH terms; Supplementary Table S2: Final curated list of associations; Supplementary Table S3: Enriched pathways following data integration; Supplementary Figure S1: Volcano plot of the differentially expressed genes for NSCLC patients with adenocarcinoma and squamous cell carcinoma; Supplementary Figure S2: Detected EVs in NSCLC patients; Supplementary Figure S3: Relative-fold change (2^−ΔΔCq^) of miR-29a-3p in EVs from early-stage NSCLC patient and healthy donors; Supplementary Figure S4: Relative-fold change (2^−ΔΔCq^) of lncRNA H19 in EVs from early-stage NSCLC patients and healthy donors; SupplementaryFile1_EVQuantificationCharacterization (https://drive.google.com/file/d/1ZFobGTmU-wo370wf1BGi11hBOcmZ1KM1/view?usp=sharing); SupplementaryFile2_RawData (https://drive.google.com/file/d/1mEKceDyZ0lsCvfg6iRu5vsTwzJAFshSQ/view?usp=sharing); SupplementaryFile3_FinalData_AnnotationLevels1_2a; SupplementaryFile4_FinalData_Comparisons; SupplementaryFile5_FinalData_Violinplots.
