# Supplementary material for: Profiling Plasma Extracellular Vesicle Metabotypes and miRNAs: An Unobserved Clue for Predicting Relapse in Patients with Early-Stage NSCLC
Source: Cancers (Basel). 2024 Nov 5;16(22):3729. doi: 10.3390/cancers16223729 (PMC11592109; doi:10.3390/cancers16223729)
Supplement: Supplementary file 1 [file cancers-16-03729-s001.zip › cancers-3233168-supplementary.pdf]

cancers

MDPI

Table S1. MeSH terms.

miRNAs in NSCLC	lncRNAs in NSCLC
MicroRNAs (D059626)	Long Noncoding RNAs (D065211)
RNA, Small Untranslated (D053686)	RNA, Untranslated (D012333)
Non-Small Cell Lung Carcinoma (D002289)	Gene Expression Regulation, Neoplastic (D005788)
Gene Expression Regulation, Neoplastic (D005788)	Non-Small Cell Lung Carcinoma (D002289)
Tumor Microenvironment (D065442)	Epigenesis, Genetic (D019934)
Extracellular Vesicles (D064796)	Extracellular Vesicles (D064796)
Biomarkers, Tumor (D015415)	Neoplasm Metastasis (D009362)
**Plasma Metabotypes of EVs in NSCLC**	
Metabolomics (D059230)	
Plasma (D010920)	
Extracellular Vesicles (D064796)	
Non-Small Cell Lung Carcinoma (D002289)	
Biomarkers, Tumor (D015415	
Liquid Biopsy (D000078677)	
Exosomes (D064797)	
Mass Spectrometry (D008442)	

Table S2. Final curated list of associations.

Genes	miRTarBase.ID	miRNAs	Gene regulation	lncRNAs
JAG1	MIRT732280	miR-26a-5p	down-regulated	AL513318.2
COL4A4	MIRT437823	miR-150-5p	up-regulated	NA
SOX2	MIRT000307	miR-145-5p	down-regulated	LINC00852
DLG1	MIRT733193	miR-9-3p	down-regulated	NA
SNAI2	MIRT007214	miR-1-3p	down-regulated	NA
SOX2	MIRT054761	miR-638	down-regulated	NA
SNAI2	MIRT007241	miR-630	down-regulated	NA
HNF1B	MIRT732483	miR-23a-3p	up-regulated	NA
JAG1	MIRT438596	miR-199a-5p	down-regulated	NA
BMP7	MIRT732128	miR-137	down-regulated	NA
CGN	MIRT004994	miR-125b-5p	up-regulated	NA
MACC1	MIRT733661	miR-497-5p	up-regulated	NA
S100A8	MIRT052953	miR-24-3p	down-regulated	NA
NTRK2	MIRT733536	miR-22-3p	down-regulated	LncRNA DGCR5
SOX2	MIRT734551	miR-450b-5p	down-regulated	NA
SNAI2	MIRT054656	miR-96-5p	down-regulated	NA
SOX2	MIRT053008	miR-340-5p	down-regulated	NA
PLD1	MIRT733430	miR-182-5p	down-regulated	NA
SOX2	MIRT005370	miR-126-3p	down-regulated	NA
SLC16A1	MIRT002665	miR-124-3p	down-regulated	NA
MMP3	MIRT735341	miR-93-5p	down-regulated	NA
JAG1	MIRT006846	miR-143-3p	down-regulated	NA
RORC	MIRT007283	miR-30b-3p	up-regulated	LINC01426
BCL11A	MIRT054585	miR-30a-5p	down-regulated	LncRNA-AC087588.2
SOX2	MIRT007184	miR-429	down-regulated	LINC00365

ARTN	MIRT006682	miR-223-3p	down-regulated	NA
SLC16A1	MIRT019664	miR-376a-3p	down-regulated	NA
SLC16A1	MIRT001745	miR-376a-5p	down-regulated	NA
MACC1	MIRT734183	miR-590-3p	up-regulated	MIR205HG
MACC1	MIRT656541	miR-200a-3p	up-regulated	NA
ABCC5	MIRT054153	miR-129-5p	down-regulated	LINC00574
MACC1	MIRT378759	miR-338-3p	up-regulated	NA
CLDN1	MIRT731836	miR-142-5p	down-regulated	NA
CLDN1	MIRT001558	miR-155-5p	down-regulated	NA
SOX2	MIRT006223	miR-34a-5p	down-regulated	NA
BCL11A	MIRT053329	miR-486-3p	down-regulated	NA
BCL11A	MIRT731890	miR-138-5p	down-regulated	NA
BMP7	MIRT005882	miR-342-3p	down-regulated	NA
BMP7	MIRT437421	miR-542-3p	down-regulated	NA
COL7A1	MIRT006382	miR-29c-3p	down-regulated	NA
SOX2	MIRT006227	miR-34c-5p	down-regulated	NA
SOX2	MIRT007030	miR-140-5p	down-regulated	NA
DDAH1	MIRT003179	miR-210-3p	up-regulated	NA
FOXA2	MIRT735287	miR-199a-3p	up-regulated	NA
MUC13	MIRT021860	miR-132-3p	up-regulated	NA
NR3C2	MIRT001167	miR-135a-5p	up-regulated	NA
NTRK2	MIRT006434	miR-200c-3p	down-regulated	NA
RAB3B	MIRT054845	miR-200b-3p	down-regulated	NA
SKP2	MIRT053122	miR-7-5p	down-regulated	NA
SNAI2	MIRT054655	miR-183-5p	down-regulated	NA
TP63	MIRT004281	miR-92a-3p	down-regulated	NA
TP63	MIRT437996	miR-301b-3p	down-regulated	NA
TRIM29	MIRT731380	miR-15b-5p	down-regulated	NA
JAG1	MIRT734232	miR-26b-5p	down-regulated	NA
CEACAM6	MIRT438274	miR-29a-3p	up-regulated	NA
NR3C2	MIRT733699	miR-548i	up-regulated	NA
RORC	MIRT734482	miR-1236-3p	up-regulated	NA
SLC6A8	MIRT731602	miR-27a-3p	down-regulated	NA
SNAI2	MIRT438640	miR-30c-5p	down-regulated	NA
TP63	MIRT005329	miR-21-5p	down-regulated	NA
FGFR2	MIRT006275	miR-19b-1-5p	down-regulated	NA
FOXA2	MIRT733743	miR-187-3p	up-regulated	NA
GOLT1A	MIRT734396	miR-378a-3p	up-regulated	NA
GPRC5A	MIRT621495	miR-103a-3p	up-regulated	NA
JAG1	MIRT006891	miR-524-5p	down-regulated	NA
JAG1	MIRT053254	miR-199b-5p	down-regulated	NA
JAG1	MIRT053289	miR-214-3p	down-regulated	NA
JAG1	MIRT732781	miR-410-3p	down-regulated	NA
KRT5	MIRT000221	miR-196a-5p	down-regulated	NA
MMP3	MIRT734847	miR-93-3p	down-regulated	NA
MUC1	MIRT005492	miR-1226-3p	up-regulated	NA
MUC1	MIRT437845	miR-455-3p	up-regulated	NA
MUC1	MIRT731255	miR-330-5p	up-regulated	NA
NTRK2	MIRT053186	miR-204-5p	down-regulated	NA

SKP2	MIRT358762	miR-3163	down-regulated	NA
SLC22A3	MIRT735451	miR-147a	up-regulated	NA
SNAI2	MIRT007240	miR-545-3p	down-regulated	NA
SNAI2	MIRT054282	miR-506-3p	down-regulated	NA
SNAI2	MIRT731608	miR-489-3p	down-regulated	NA
SNAI2	MIRT735545	miR-497-3p	down-regulated	NA
SOX2	MIRT005692	miR-522-3p	down-regulated	NA
SOX2	MIRT006226	miR-34b-3p	down-regulated	NA
SOX2	MIRT021924	miR-128-3p	down-regulated	NA
SOX2	MIRT054380	miR-625-5p	down-regulated	NA
SOX2	MIRT438929	miR-1181	down-regulated	NA
SOX2	MIRT732041	miR-146a-5p	down-regulated	NA
SOX2	MIRT733145	miR-371a-5p	down-regulated	NA
TFPI2	MIRT005774	miR-616-3p	up-regulated	NA
TFRC	MIRT003744	miR-320a	down-regulated	NA
TP63	MIRT004248	miR-203a-3p	down-regulated	NA
TRIM29	MIRT018621	miR-335-5p	down-regulated	NA
TRIM29	MIRT731749	miR-185-5p	down-regulated	NA
VSNL1	MIRT003036	miR-181b-5p	down-regulated	NA

Table S3. Enriched pathways following data integration.

Pathway	Total number of compounds	Statistic Q-value	Expected Q-value	Raw p-value	Holm p-value	FDR	miRNA	Database	Adjusted p-value
**Glycerophospholipidmetabolism**	**36**	**10.789**	**2.439**	**0.034**	**1**	**0.160**	**miR-338-3p**	**KEGG**	**0.024**
**Alanine, aspartate andglutamatemetabolism**	**28**	**9.356**	**2.439**	**0.049**	**1**	**0.160**	**miR-29a-3p**	**KEGG**	**0.028**
Purinemetabolism	65	3.241	2.439	0.252	1	0.347	miR-338-3p	KEGG	0.041
Cysteineandmethioninemetabolism	33	2.888	2.439	0.303	1	0.388	miR-29a-3p	KEGG	0.021
Cysteineandmethioninemetabolism	33	2.888	2.439	0.303	1	0.388	miR-200b-3p	KEGG	0.005
Glycolysis, gluconeogenesis	26	0.006	2.439	0.962	1	0.962	miR-338-3p	KEGG	0.024
Pyruvatemetabolism	22	0.006	2.439	0.962	1	0.962	miR-338-3p	KEGG	0.014
Pyruvate metabolism	22	0.006	2.439	0.962	1	0.962	miR-128-3p	REACTOME	0.036

The table summarises the enriched pathways obtained after integrating untargeted metabolomics (Metabolite Set Enrichment Analysis) and miRNA (miRPathDB) datasets. FDR: False Discovery rate.

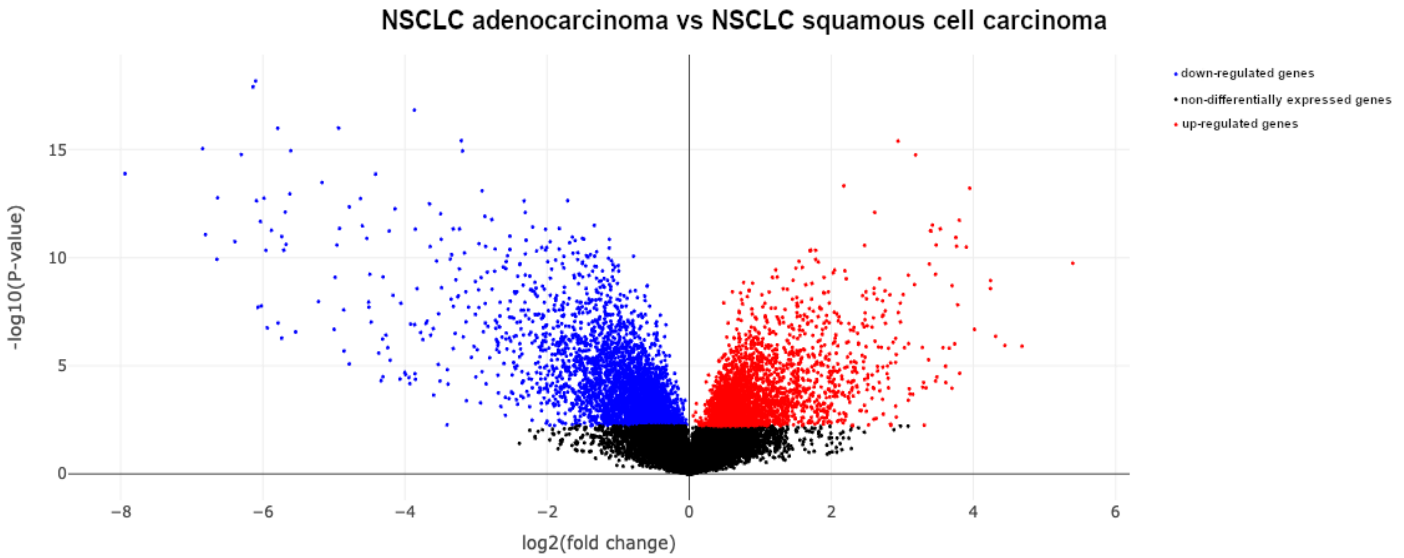

Figure S1. Volcano plot of the differentially expressed genes for NSCLC patients with adenocarcinoma and squamous cell carcinoma. Each point represents a gene. Blue dots indicate significantly down-regulated genes. Red dots represent significantly up-regulated genes (adjusted p-value < 0.05). Black dots correspond to non-differentially expressed genes. The x-axis represents log2 (fold change), while the y-axis represents -log10 (p-value), illustrating the magnitude and significance of gene expression differences.

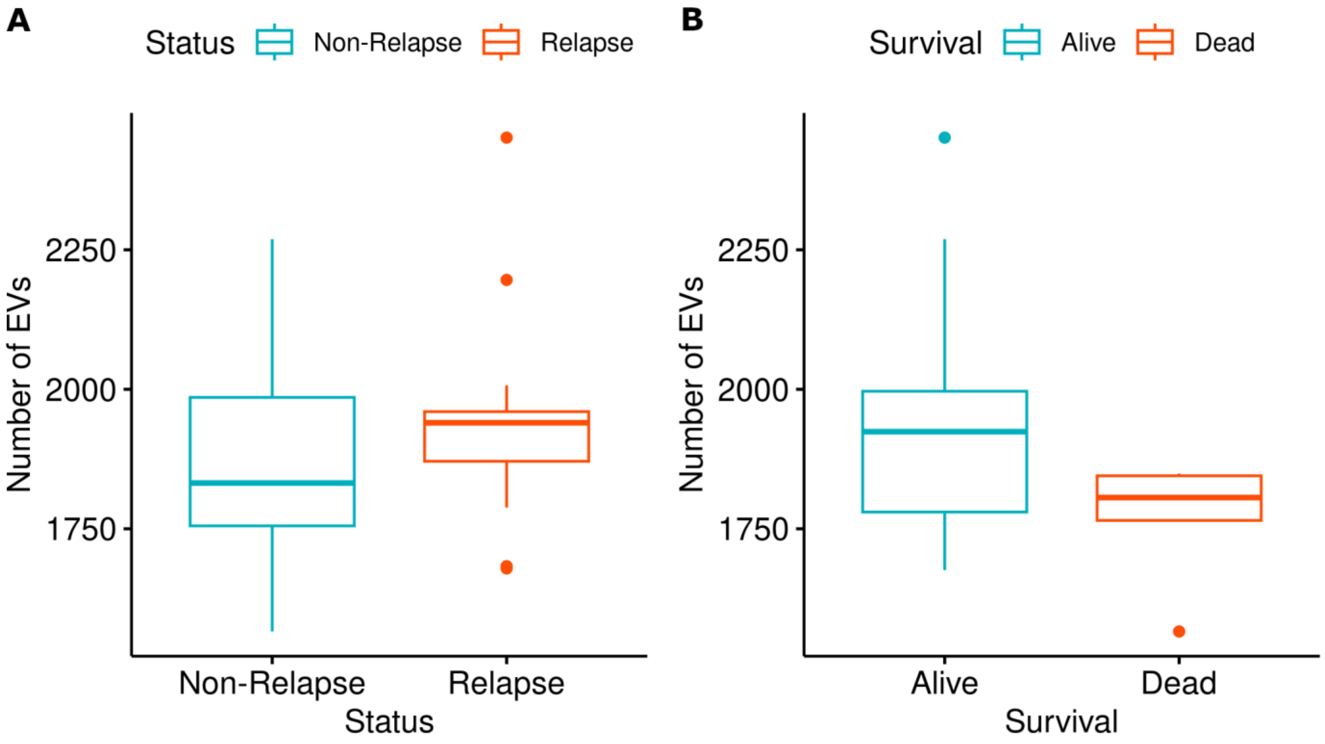

Figure S2. Detected EVs in NSCLC patients. Boxplots illustrate the distribution of detected EVs in samples of early-stage NSCLC patients, categorized by A) relapse and B) overall survival. The y-axis indicates the number of EVs detected in each sample, while the x-axis indicates the disease progression status or patient overall survival, respectively. Statistical analysis revealed no significant difference in EV counts when disease progression (*p*-value = 0.280) and patient overall survival (*p*-value = 0.093) were considered.

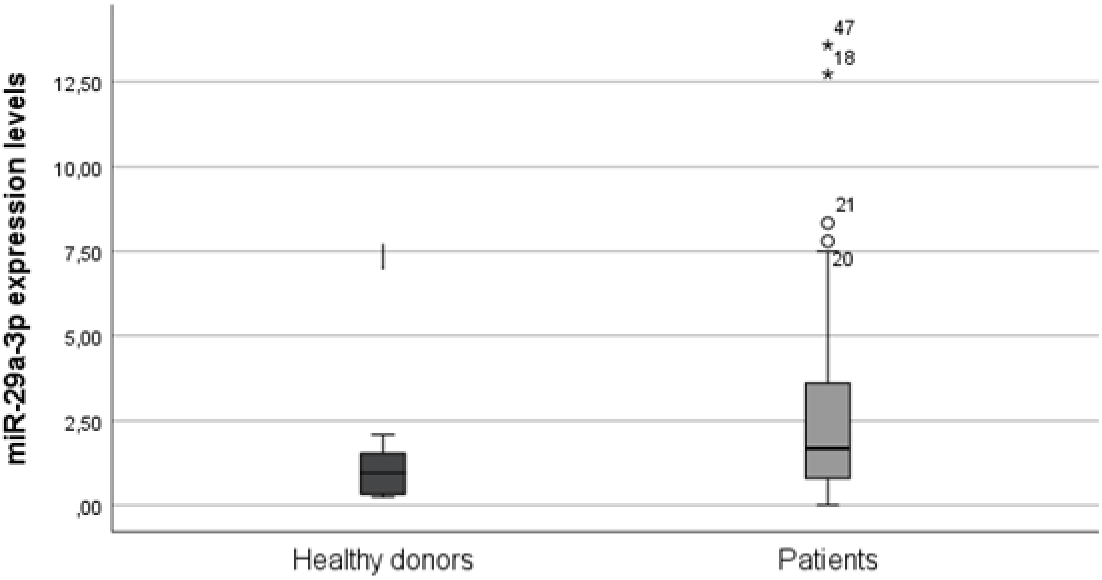

Figure S3. Relative fold change ($2^{-\Delta\Delta Cq}$) of *miR-29a-3p* in EVs from early-stage NSCLC patient and Healthy donors.

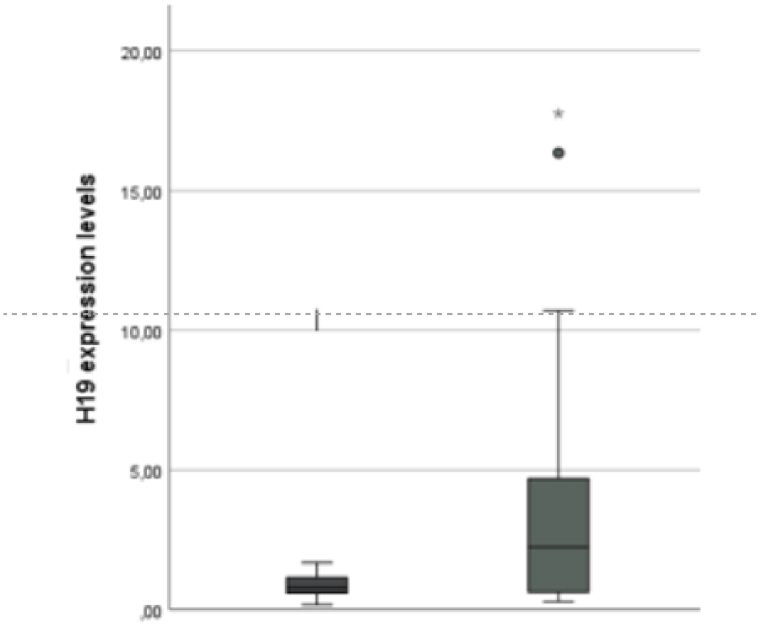

Figure S4. Relative fold change ($2^{-\Delta\Delta Cq}$) of *lncRNA H19* in EVs from early-stage NSCLC patients and Healthy donors.

Supplementary methods

Isolation of extracellular vesicles – benchmarking by liquid chromatography-tandem mass spectrometry

Aiming to benchmark and verify further our reference framework, this approach serves to validate the methods and parameters used in the main analysis, ensuring robust and reproducible results. To confirm the presence of protein markers (to name but a few, CD63, CD81, and TSG101), EV proteins were extracted by lysing EVs in RIPA buffer, followed by protein quanti-fication using a bicinchoninic acid (BCA) assay. The protein samples were reduced with

dithiothreitol (DTT), alkylated with iodoacetamide, and digested overnight with trypsin. The resulting peptides were desalted using C18 solid-phase extraction and dried using a vacuum concentrator. Peptide samples were resuspended in 0.1% formic acid and ana-lysed using liquid chromatography-tandem mass spectrometry on a UPLC/HPLC - LTQ Velos Orbitrap mass spectrometer (Thermo Fisher Scientific) upon their separation on a reverse-phase C18 column. Data-dependent acquisition (DDA) was employed, selecting the 10 most intense precursor ions for fragmentation. Raw data were processed using MaxQuant software for protein identification and quantification. Searches were conducted against the UniProt database with a false discovery rate (FDR) set to <1%. Label-free quantification was applied to compare protein abundance across samples.

Quantification and characterization of EVs

Our pipeline designed to identify miRNAs and lncRNAs that can serve as candidate biomarkers for NSCLC is described below. For gene expression analysis, GSE10245(Gene Expression Omnibus, GEO) isselected using the GEO2R online tool. The GEO2R platform can be accessed by clicking the "Analyze with GEO2R" button on the dataset page, which opens the interface, ready for analysis. A direct link for analysing the dataset is provided here. In GEO2R, samples are divided into two groups: adenocarcinoma and squamous cell carcinoma, based on the "Disease state" column annotation. Once the groups are defined and the analysis is completed, the results can be downloaded and further analysed in RStudio.To analyze the results in RStudio, follow the steps outlined below:

Set Working Directory and Unzip Data

```
# Define the list of required packages
required_packages <-c("readxl", "xlsx", "dplyr", "readr")
# Function to check and install missing packages required for the analysis
install_if_missing <-function(packages) {
for (pkg in packages) {
if (!requireNamespace(pkg, quietly =TRUE)) {
message(paste("Package", pkg, "not found. Installing now..."))
install.packages(pkg)
    } else {
message(paste("Package", pkg, "is already installed."))
    }
  }
}

install_if_missing(required_packages)

## Package readxl is already installed.

## Package xlsx is already installed.

## Package dplyr is already installed.

## Package readr is already installed.
```

```r
# Set working directory
working_dir <-"~/R_workplace/tmp_NSCLC/NSCLC/Reproduction_test/"
setwd(working_dir)
# Unzip the file containing necessary data files
unzip(paste0(working_dir, "Data.zip"), exdir =paste0(working_dir, "Data"))
# Check if the "Results" directory exists
if (!dir.exists("Results")) {
# Create the "Results" directory if it doesn't exist
dir.create("Results")
cat("Directory 'Results' created.\n")
} else {
cat("Directory 'Results' already exists.\n")
}

## Directory 'Results' created.
```

The following files are required for the analysis and should be in the Data directory: GSE10245.top.table.tsv, lnctard2.0.txt, and miRTarBase_SE_WR.xls

Load Gene Expression Data

The file GSE10245.top.table.tsv contains the differential gene expression results obtained from GEO2R (data acquisition date: 28 September 2022). To load and filter the data, use the following code:

```r
# Load GEO2R output and filter for significant changes
gene_data <-read.table('Data/GSE10245.top.table.tsv', sep ='\t', header =TRUE)
## Warning in scan(file = file, what = what, sep = sep, quote = quote, dec = dec,
## : EOF within quoted string
filtered_genes <- gene_data[which(gene_data$adj.P.Val <0.05&abs(gene_data$logFC) >2),
 ]
head(filtered_genes)
##            ID adj.P.Val  P.Value         t        B logFC Gene.symbol
## 1 206033_s_at  3.42e-14 6.69e-19 -13.16729 32.08893 -6.10        DSC3
## 2   206032_at  3.42e-14 1.25e-18 -12.97677 31.50776 -6.14        DSC3
## 3   244107_at  2.66e-13 1.46e-17 -12.24007 29.21093 -3.87        DSC3
## 4 211194_s_at  1.13e-12 1.02e-16 -11.67365 27.39170 -4.93        TP63
## 5 206166_s_at  1.13e-12 1.03e-16 -11.66934 27.37769 -5.79       CLCA2
## 6   206156_at  3.14e-12 3.89e-16 -11.28801 26.12701 -3.21        GJB5
##                       Gene.title
## 1                  desmocollin 3
## 2                  desmocollin 3
## 3                  desmocollin 3
## 4              tumor protein p63
## 5 chloride channel accessory 2
## 6  gap junction protein beta 5
gene_symbols <-unique(filtered_genes$Gene.symbol)
```

Load miRNA Data and Filter for Human Interactions

The file miRTarBase_SE_WR.xls contains miRNA-gene interactions backed by strong experimental evidence (data acquisition date: 23 January 2022). First, load the data and filter it for human miRNA interactions:

```
# Load miRNA interaction data
library(readxl)
mirna_data <-read_xls("Data/miRTarBase_SE_WR.xls")
# Filter for human-related interactions
filtered_mirna_data <- mirna_data[mirna_data$`Species (miRNA)`=='Homo sapiens', ]
write.table(filtered_mirna_data, 'Data/miRTarBase_Human_Strong.txt')
```

Identify miRNA-Gene Interactions

Next, match the differentially expressed genes with miRNA targets:

```
# Read filtered miRNA data
miRNA_targets <-read.table('Data/miRTarBase_Human_Strong.txt')
# Find the interactions for genes of interest
idx <-unlist(lapply(gene_symbols, function(g) which(miRNA_targets$Target.Gene == g)))
selected_miRNA_data <- miRNA_targets[idx, ]
selected_miRNA_data <- selected_miRNA_data[!duplicated(selected_miRNA_data$miRNA), ]
```

Match Gene Regulation Direction

We match the fold change direction (up-regulated or down-regulated) for each selected gene:

```
# Identify the direction of regulation (up or down)
gene_changes <-unique(selected_miRNA_data$Target.Gene)
idx2 <-unlist(lapply(gene_changes, function(g) which(filtered_genes$Gene.symbol ==
g)))
gene_regulation <- filtered_genes[idx2, ]
gene_regulation <- gene_regulation[!duplicated(gene_regulation$Gene.symbol), ]

regulation_matrix <-data.frame(genes = gene_regulation$Gene.symbol,
regulation =ifelse(gene_regulation$logFC >0, 'up-regulated', 'down-regulated'))

# Merge miRNA data with regulation info
colnames(selected_miRNA_data)[4] <-"genes"
selected_miRNA_data <-merge(selected_miRNA_data, regulation_matrix, by ="genes")
head(selected_miRNA_data)
```

```
##    genes miRTarBase.ID          miRNA Species..miRNA. Target.Gene..Entrez.ID.
## 1  ABCC5    MIRT054153 hsa-miR-129-5p    Homo sapiens                   10057
## 2   ARTN    MIRT006682 hsa-miR-223-3p    Homo sapiens                    9048
## 3 BCL11A    MIRT053329 hsa-miR-486-3p    Homo sapiens                   53335
## 4 BCL11A    MIRT054585 hsa-miR-30a-5p    Homo sapiens                   53335
## 5 BCL11A    MIRT731890 hsa-miR-138-5p    Homo sapiens                   53335
## 6   BMP7    MIRT005882 hsa-miR-342-3p    Homo sapiens                     655
```

```
##   Species..Target.Gene.
## 1          Homo sapiens
## 2          Homo sapiens
## 3          Homo sapiens
## 4          Homo sapiens
## 5          Homo sapiens
## 6          Homo sapiens
##
                    Experiments
## 1                                            Immunohistochemistry//Luciferase reporter as
say//Microarray//qRT-PCR//Western blot
## 2                                                                              Luciferase re
porter assay//qRT-PCR//Western blot
## 3                                                                              Luciferase re
porter assay//qRT-PCR//Western blot
## 4                                                                                      Lucif
erase reporter assay//Western blot
## 5 Luciferase reporter assay//Next Generation Sequencing (NGS)//qRT-PCR//RNA-binding
protein immunoprecipitation//Western blot
## 6                                                                             Luciferase rep
orter assay//Northern blot//qRT-PCR
##     Support.Type References..PMID.    regulation
## 1 Functional MTI          25344911 down-regulated
## 2 Functional MTI          21453483 down-regulated
## 3 Functional MTI          23593217 down-regulated
## 4 Functional MTI          23758992 down-regulated
## 5 Functional MTI          28303002 down-regulated
## 6 Functional MTI          21172025 down-regulated
```

Export Preliminary miRNA Data

The preliminary miRNA data is exported to an Excel file for further exploration:

```
# Export miRNA data to Excel
library(xlsx)
xlsx::write.xlsx(selected_miRNA_data, 'Data/Preliminary_miRNA.xlsx',
sheetName ="Sheet1", col.names =TRUE, row.names =FALSE)
```

Load and Filter lncRNA Data

The file lnctard2.0.txt contains lncRNA-target regulations (data acquisition date: 19 October 2022). We filter it for entries related to NSCLC:

```
# Load lncRNA data
lncRNA_data <-read.delim('Data/lnctard2.0.txt')
## Warning in scan(file = file, what = what, sep = sep, quote = quote, dec = dec,
##: EOF within quoted string
```

```
# Filter for NSCLC-related interactions
nsclc_lncRNA_data <- lncRNA_data[lncRNA_data$DiseaseName =='Non-small cell lung cancer
', ]
xlsx::write.xlsx(nsclc_lncRNA_data, 'Data/Preliminary_lncRNA.xlsx',
sheetName ="Sheet1", col.names =TRUE, row.names =FALSE)
```

MiRNA Pathways Across Databases

To identify the target pathways of the miRNAs, we utilized the miRPathDB 2.0 database after we retrieved the "miRPathDB2_hsa_p_values.tar.gz" archive (data acquisition date: 29 November 2022). This archive contains pathway-by-miRNA matrices for each database along with their confidence levels. Each matrix (in CSV format) provides FDR-adjusted p-values for miRNA-pathway pairs, where rows correspond to miRNAs and columns represent individual pathways. These matrices are generated based on the GeneTrail2 enrichment analysis results. The archive is decompressed, and only the CSV files containing strongly validated experimental data are selected for further analysis. Herein, we investigated the pathways targeted by the miRNAs in our ranked list across multiple databases, including WikiPathways, Reactome, KEGG, and GO.

```
# Unzip the file containing necessary data files
unzip(paste0(working_dir, "hsa.zip"), exdir =paste0(working_dir, "hsa"))
# Load pathway with strong experimental validation for the miRNA in the pathway
wiki=read.csv("hsa/hsa/WIKIPATHWAYS_validated_miRTarBase_strong.csv")
reactome=read.csv("hsa/hsa/REACTOME_validated_miRTarBase_strong.csv")
kegg=read.csv("hsa/hsa/KEGG_validated_miRTarBase_strong.csv")
go_mf=read.csv("hsa/hsa/GO_MF_validated_miRTarBase_strong.csv")
go_cc=read.csv("hsa/hsa/GO_CC_validated_miRTarBase_strong.csv")
go_bp=read.csv("hsa/hsa/GO_BP_validated_miRTarBase_strong.csv")
all_databases=list(wiki,reactome,kegg,go_mf,go_cc,go_bp)
names(all_databases)=c('wiki','reactome','kegg','go_mf','go_cc','go_bp')
```

Identify the pathways related to the manually curated ranked list

The manually curated ranked list is provided in Supplementary Table 1.

```
# Load the ranked list provided
ranked_dat<-read_excel("NSCLC_miRNA_Ranking_LncRNA.xlsx")
# initialize data.frames
my_mat=matrix(NA,ncol =3,nrow =1)
my_mat=as.data.frame(my_mat)
my_mat2=matrix(NA,ncol =3,nrow =1)
my_mat2=as.data.frame(my_mat2)
n=0
# scan each database for match
for (i inc(1:dim(ranked_dat)[1])) {  # our miRNA
print(paste('miRNA:',i))
for (i1 inc(1:length(all_databases))) { # database id
for (i2 inc(1:dim(all_databases[[i1]])[1])) { # index in database
```

```
      tmp_idx=which(ranked_dat$miRNA[i]==rownames(all_databases[[i1]]))

if(!rlang::is_empty(tmp_idx)){
        n=n+1
        tmp_mat=matrix(c(i,i2,tmp_idx),byrow = T,nrow =1,ncol =3)
        my_mat[n,]=tmp_mat
        tmp_mat2=matrix(c(ranked_dat$miRNA[i],names(all_databases)[i1],tmp_idx),byrow =
 T,nrow =1,ncol =3)
        my_mat2[n,]=tmp_mat2
      }

    }
  }
}

## [1] "miRNA: 1"
## [1] "miRNA: 2"
## [1] "miRNA: 3"
## [1] "miRNA: 4"
## [1] "miRNA: 5"
## [1] "miRNA: 6"
## [1] "miRNA: 7"
## [1] "miRNA: 8"
## [1] "miRNA: 9"
## [1] "miRNA: 10"
## [1] "miRNA: 11"
## [1] "miRNA: 12"
## [1] "miRNA: 13"
## [1] "miRNA: 14"
## [1] "miRNA: 15"
## [1] "miRNA: 16"
## [1] "miRNA: 17"
## [1] "miRNA: 18"
## [1] "miRNA: 19"
## [1] "miRNA: 20"
## [1] "miRNA: 21"
## [1] "miRNA: 22"
## [1] "miRNA: 23"
## [1] "miRNA: 24"
## [1] "miRNA: 25"
## [1] "miRNA: 26"
## [1] "miRNA: 27"
## [1] "miRNA: 28"
```

```
## [1] "miRNA: 29"
## [1] "miRNA: 30"
## [1] "miRNA: 31"
## [1] "miRNA: 32"
## [1] "miRNA: 33"
## [1] "miRNA: 34"
## [1] "miRNA: 35"
## [1] "miRNA: 36"
## [1] "miRNA: 37"
## [1] "miRNA: 38"
## [1] "miRNA: 39"
## [1] "miRNA: 40"
## [1] "miRNA: 41"
## [1] "miRNA: 42"
## [1] "miRNA: 43"
## [1] "miRNA: 44"
## [1] "miRNA: 45"
## [1] "miRNA: 46"
## [1] "miRNA: 47"
## [1] "miRNA: 48"
## [1] "miRNA: 49"
## [1] "miRNA: 50"
## [1] "miRNA: 51"
## [1] "miRNA: 52"
## [1] "miRNA: 53"
## [1] "miRNA: 54"
## [1] "miRNA: 55"
## [1] "miRNA: 56"
## [1] "miRNA: 57"
## [1] "miRNA: 58"
## [1] "miRNA: 59"
## [1] "miRNA: 60"
## [1] "miRNA: 61"
## [1] "miRNA: 62"
## [1] "miRNA: 63"
## [1] "miRNA: 64"
## [1] "miRNA: 65"
## [1] "miRNA: 66"
## [1] "miRNA: 67"
## [1] "miRNA: 68"
## [1] "miRNA: 69"
## [1] "miRNA: 70"
```

```
## [1] "miRNA: 71"
## [1] "miRNA: 72"
## [1] "miRNA: 73"
## [1] "miRNA: 74"
## [1] "miRNA: 75"
## [1] "miRNA: 76"
## [1] "miRNA: 77"
## [1] "miRNA: 78"
## [1] "miRNA: 79"
## [1] "miRNA: 80"
## [1] "miRNA: 81"
## [1] "miRNA: 82"
## [1] "miRNA: 83"
## [1] "miRNA: 84"
## [1] "miRNA: 85"
## [1] "miRNA: 86"
## [1] "miRNA: 87"
## [1] "miRNA: 88"
## [1] "miRNA: 89"
## [1] "miRNA: 90"
## [1] "miRNA: 91"
## [1] "miRNA: 92"
## [1] "miRNA: 93"
```

```
my_mat3=my_mat2[!duplicated(my_mat2[c(1:3)]),]
my_mat3$V3=as.numeric(my_mat3$V3)

saveRDS(my_mat2,"Results/miRNA_pathways_matches.rds")
# get the pathway names and p-values for the identified miRNAs
matched_pathways=matrix(NA,ncol =4,nrow =1)
matched_pathways=as.data.frame(matched_pathways)
colnames(matched_pathways)=c('miRNA','Database','Paths','p_adjusted')
for (i1 inc(1:dim(my_mat3)[1])) {
  tmp=all_databases[[my_mat3$V2[i1]]][my_mat3$V3[i1],which(all_databases[[my_mat3$V2[i
1]]][my_mat3$V3[i1],]<0.05)]
  get_cols=colnames(all_databases[[my_mat3$V2[i1]]])[which(all_databases[[my_mat3$V2[i
1]]][my_mat3$V3[i1],]<0.05)]
if(is.null(dim(tmp)) ){
names(tmp)=get_cols
    tmp2=t(tmp)
    path_names=colnames(tmp2)
  }else{
    tmp2=t(tmp)
```

```
    path_names=rownames(tmp2)
  }
  tmp2=cbind(path_names,tmp2)
  tmp2=as.data.frame(tmp2)
colnames(tmp2)=c('Paths','p_adjusted')
  my_mir=rep(my_mat3$V1[i1],dim(tmp2)[1])
  my_db=rep(my_mat3$V2[i1],dim(tmp2)[1])
  tmp3=cbind(my_mir,my_db,tmp2)
colnames(tmp3)=c('miRNA','Database','Paths','p_adjusted')
  matched_pathways=rbind(matched_pathways,tmp3)
rownames(matched_pathways)=NULL
}
matched_pathways=na.omit(matched_pathways)
matched_pathways$Paths=tolower(matched_pathways$Paths)
colnames(matched_pathways)[3] <-"Pathway"
write.xlsx(matched_pathways, file='Results/miRNA_Pathways.xlsx', sheetName ="Sheet1",
col.names = T, row.names = F, append =FALSE)
```

Merge and Export Results

Merge the miRNA-pathway matches with metabolite pathway data and export the results:

```
library(readr)
# Load metabolite pathway data
metab_data <-read_csv("msea_qea_result_treatment.csv")
## New names:
## Rows: 32 Columns: 8
## ── Column specification
## ──────────────────────────────────────────────────────── Delimiter: "," chr
## (1): ...1 dbl (7): Total Cmpd, Hits, Statistic Q, Expected Q, Raw p, Holm p,
## FDR
## ℹ Use `spec()` to retrieve the full column specification for this data. ℹ
## Specify the column types or set `show_col_types = FALSE` to quiet this message.
## • `` -> `...1`
colnames(metab_data)[1] <-"Pathway"
metab_data$Pathway <-tolower(gsub("[^a-zA-Z0-9]", ".", metab_data$Pathway))
# Merge and export final pathway matches
merged_table <-merge(metab_data, matched_pathways, by ="Pathway")
sorted_data <- merged_table[order(merged_table$`Raw p`, decreasing = F), ]
print(sorted_data)
```

```
##                                      Pathway Total Cmpd Hits Statistic Q
## 4              glycerophospholipid.metabolism         36    1  10.7890000
## 1 alanine..aspartate.and.glutamate.metabolism         28    1   9.3562000
## 6                          purine.metabolism         65    2   3.2413000
```

```
## 2          cysteine.and.methionine.metabolism        33    2   2.8882000
## 3          cysteine.and.methionine.metabolism        33    2   2.8882000
## 5                glycolysis...gluconeogenesis        26    1   0.0059106
## 7                        pyruvate.metabolism        22    1   0.0059106
## 8                        pyruvate.metabolism        22    1   0.0059106
##   Expected Q    Raw p Holm p     FDR          miRNA Database p_adjusted
## 4      2.439 0.033691      1 0.16003  hsa-miR-338-3p     kegg  0.0237237
## 1      2.439 0.048838      1 0.16003  hsa-miR-29a-3p     kegg  0.0279974
## 6      2.439 0.251530      1 0.34714  hsa-miR-338-3p     kegg   0.041031
## 2      2.439 0.303350      1 0.38829  hsa-miR-29a-3p     kegg  0.0217841
## 3      2.439 0.303350      1 0.38829 hsa-miR-200b-3p     kegg 0.00460199
## 5      2.439 0.961460      1 0.96146  hsa-miR-338-3p     kegg   0.024062
## 7      2.439 0.961460      1 0.96146  hsa-miR-338-3p     kegg  0.0136996
## 8      2.439 0.961460      1 0.96146  hsa-miR-128-3p reactome  0.0356303
```

```
xlsx::write.xlsx(sorted_data, 'Results/miRNA_Pathways.xlsx',
sheetName ="Sheet1", col.names =TRUE, row.names =FALSE)
```
