# Supplementary material for: Profiling Plasma Extracellular Vesicle Metabotypes and miRNAs: An Unobserved Clue for Predicting Relapse in Patients with Early-Stage NSCLC
Source: Cancers (Basel). 2024 Nov 5;16(22):3729. doi: 10.3390/cancers16223729 (PMC11592109; doi:10.3390/cancers16223729)
Supplement: Supplementary file 1 [file cancers-16-03729-s001.zip › SupplementaryFile5_FinalData_Violinplots.pdf]

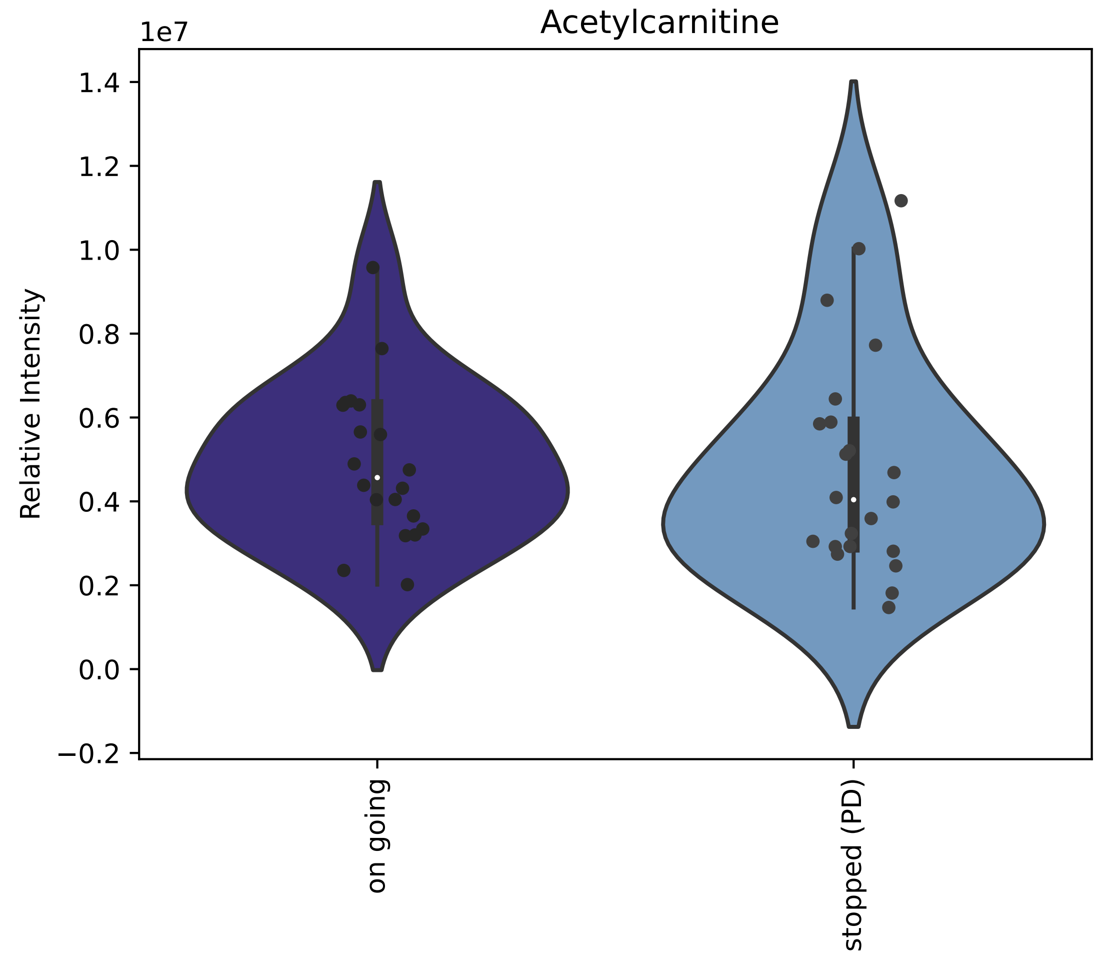
Acetylcarnitine
1e7
Relative Intensity
1.4
1.2
1.0
0.8
0.6
0.4
0.2
0.0
−0.2
on going
stopped (PD)

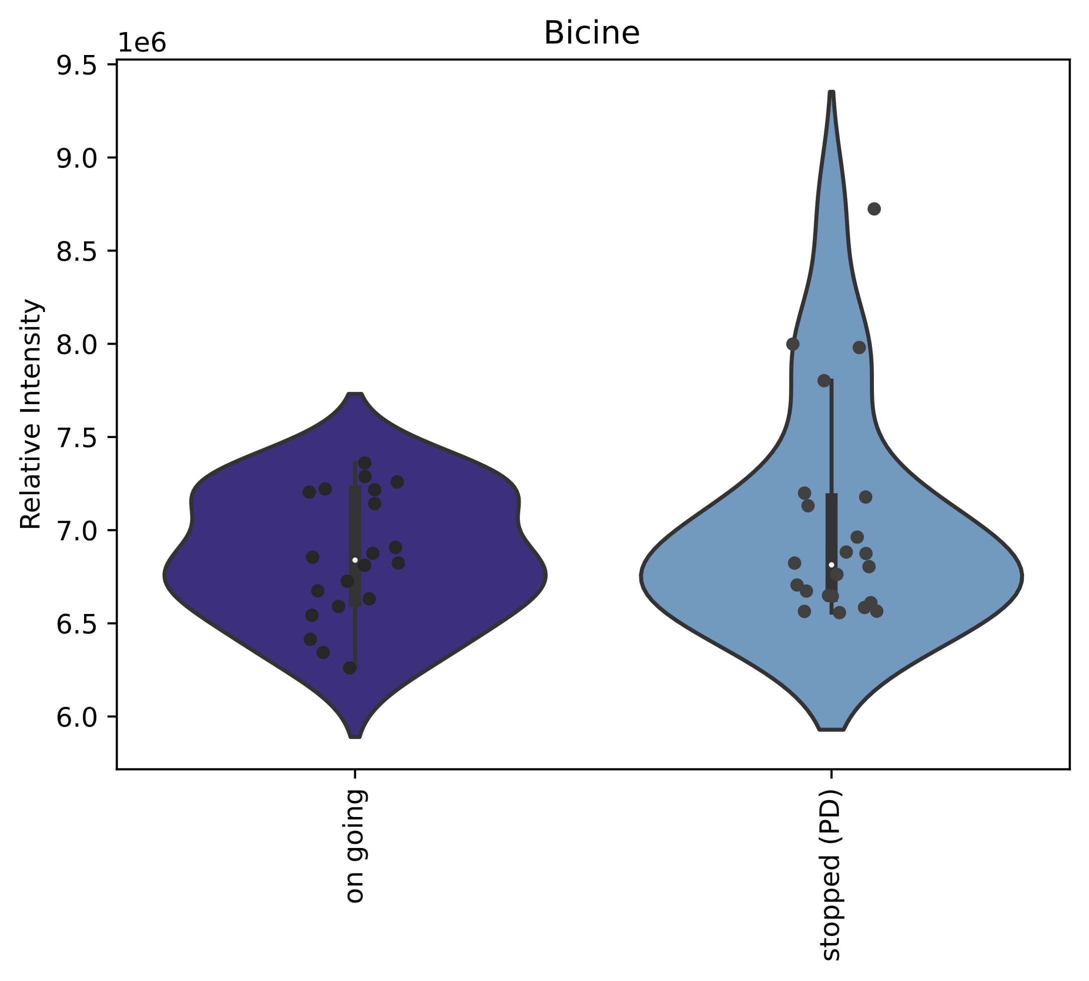

Bicine
1e6
9.5
9.0
8.5
8.0
7.5
7.0
6.5
6.0
Relative Intensity
on going
stopped (PD)

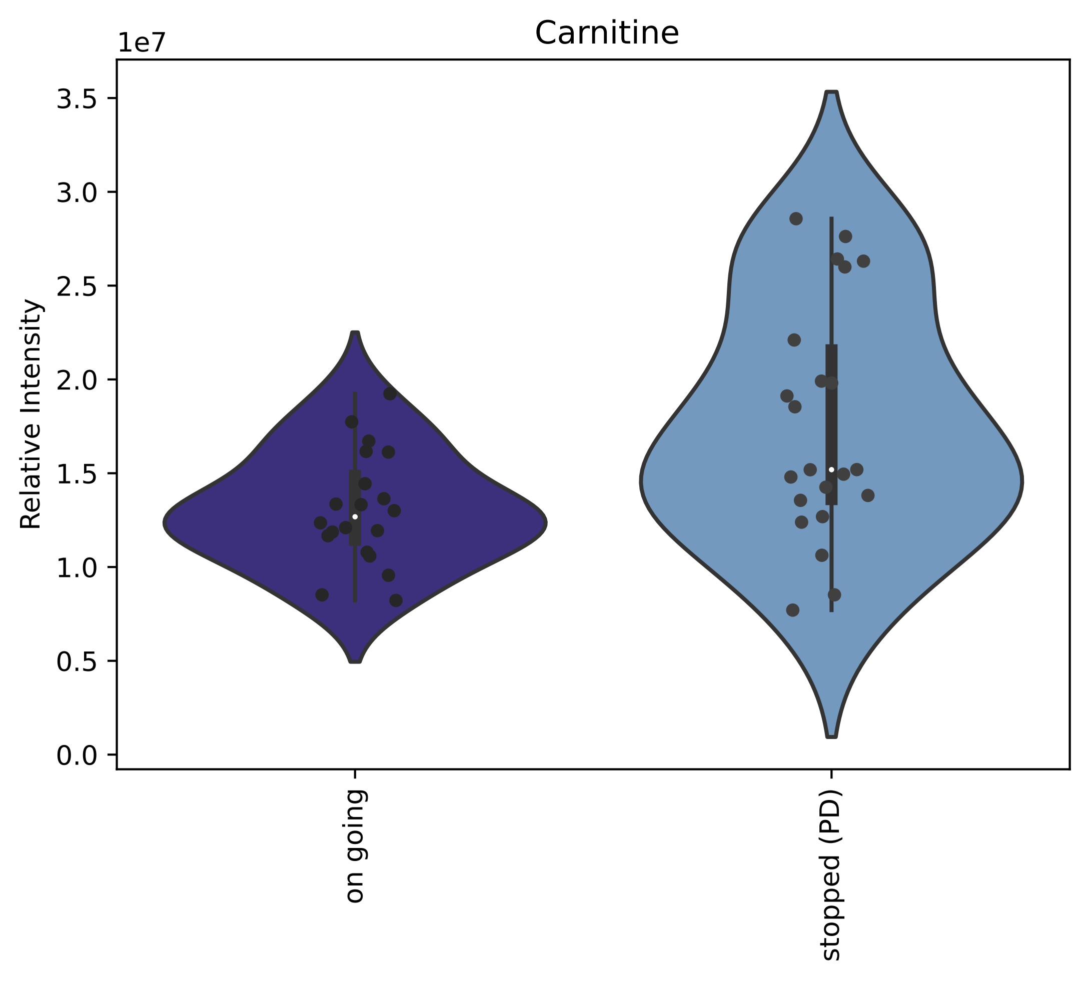

Carnitine
1e7
3.5
3.0
2.5
2.0
1.5
1.0
0.5
0.0
Relative Intensity
on going
stopped (PD)

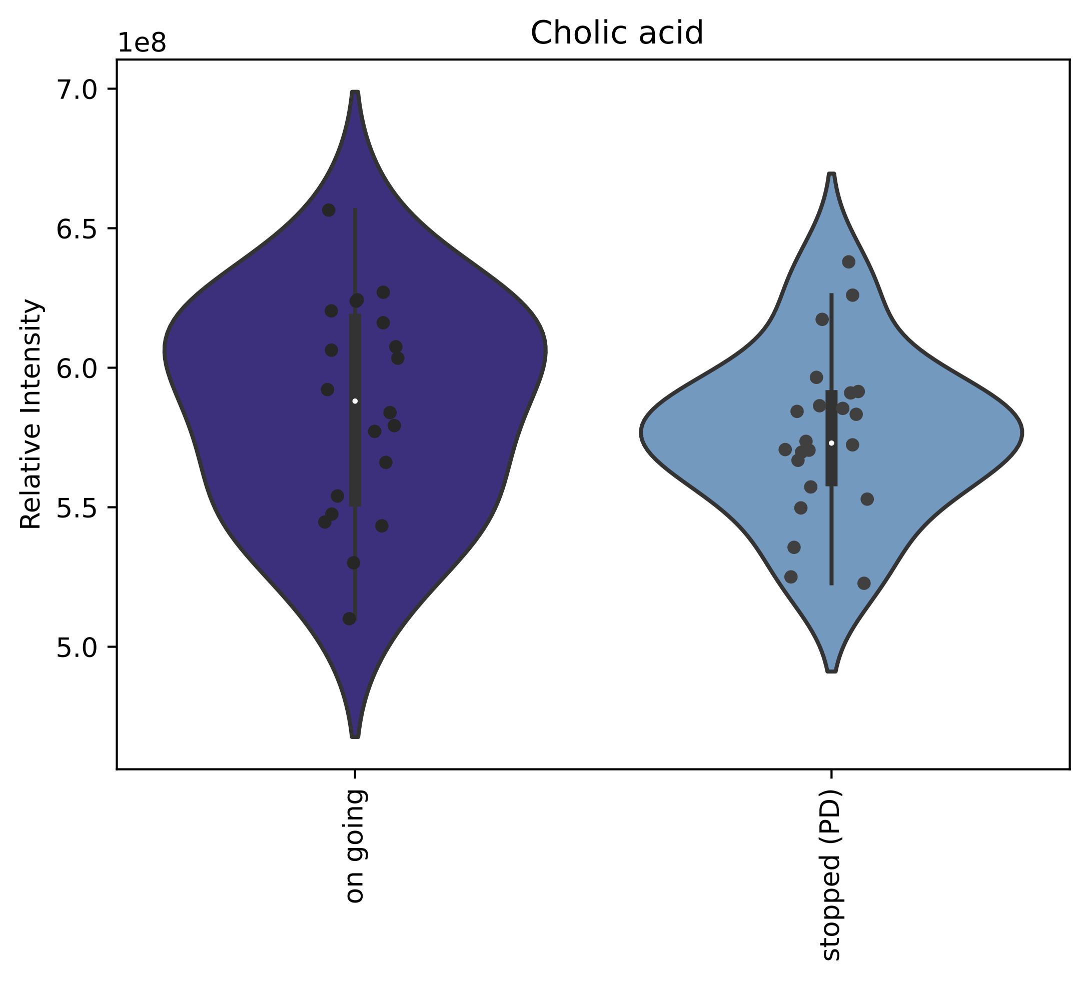
Cholic acid
1e8
7.0
6.5
6.0
5.5
5.0
Relative Intensity
on going
stopped (PD)

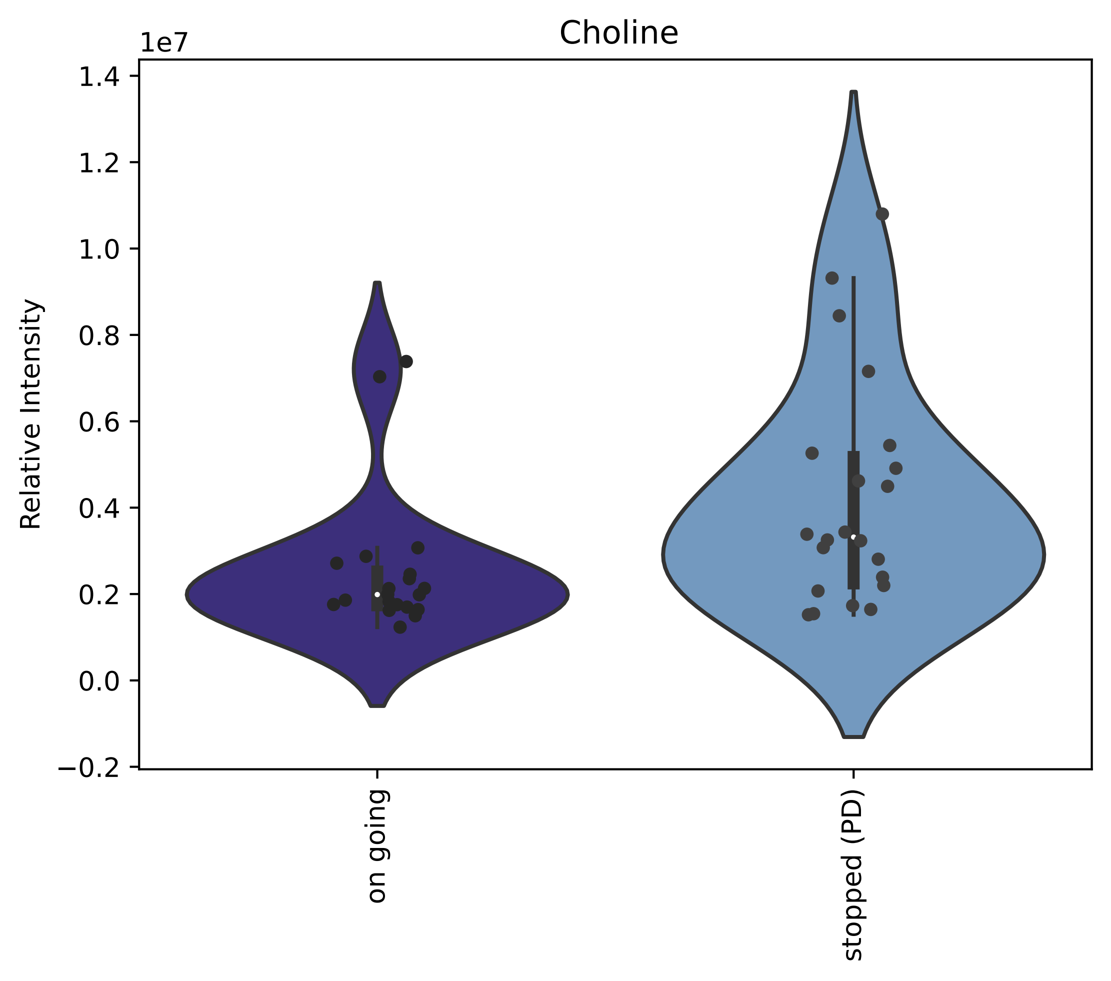

Choline
1e7
1.4
1.2
1.0
0.8
0.6
0.4
0.2
0.0
−0.2
Relative Intensity
on going
stopped (PD)

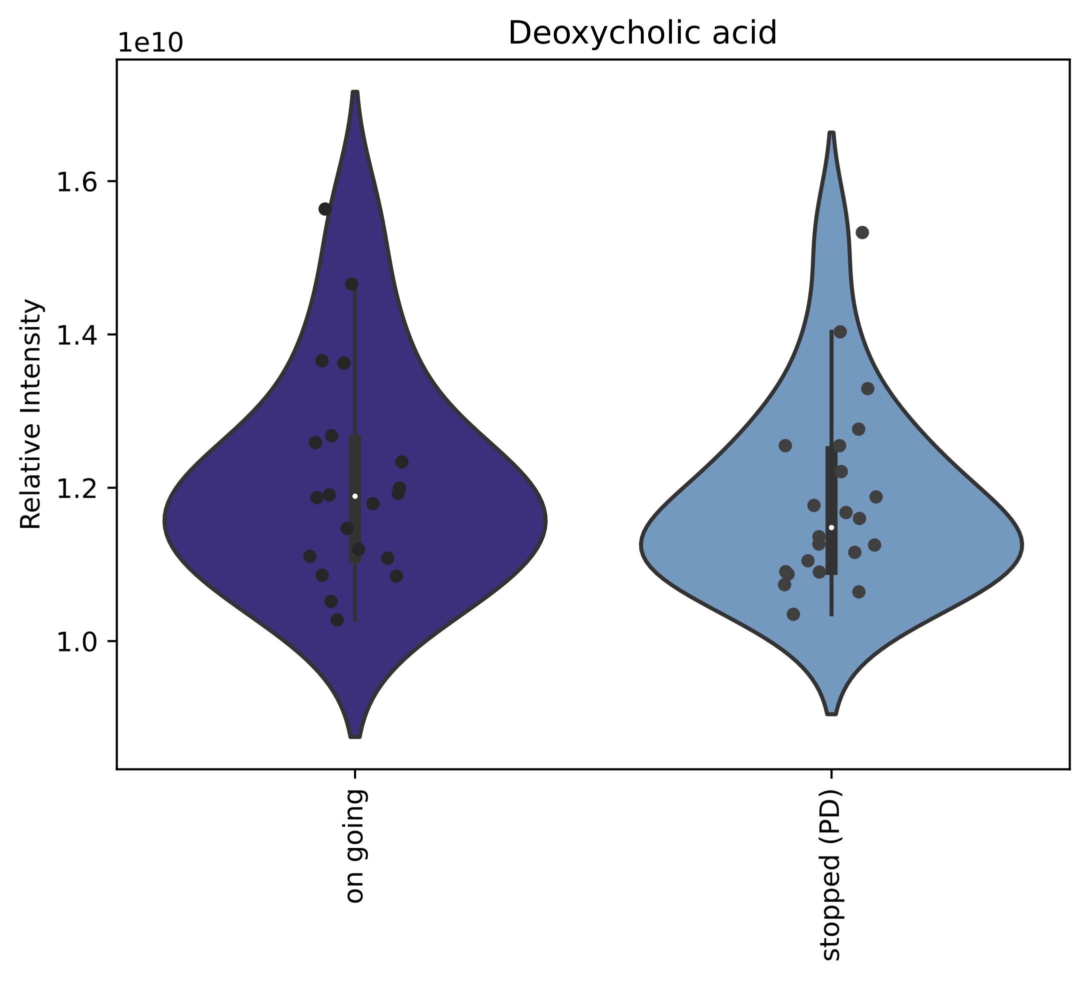
Deoxycholic acid
1e10
1.6
1.4
1.2
1.0
Relative Intensity
on going
stopped (PD)

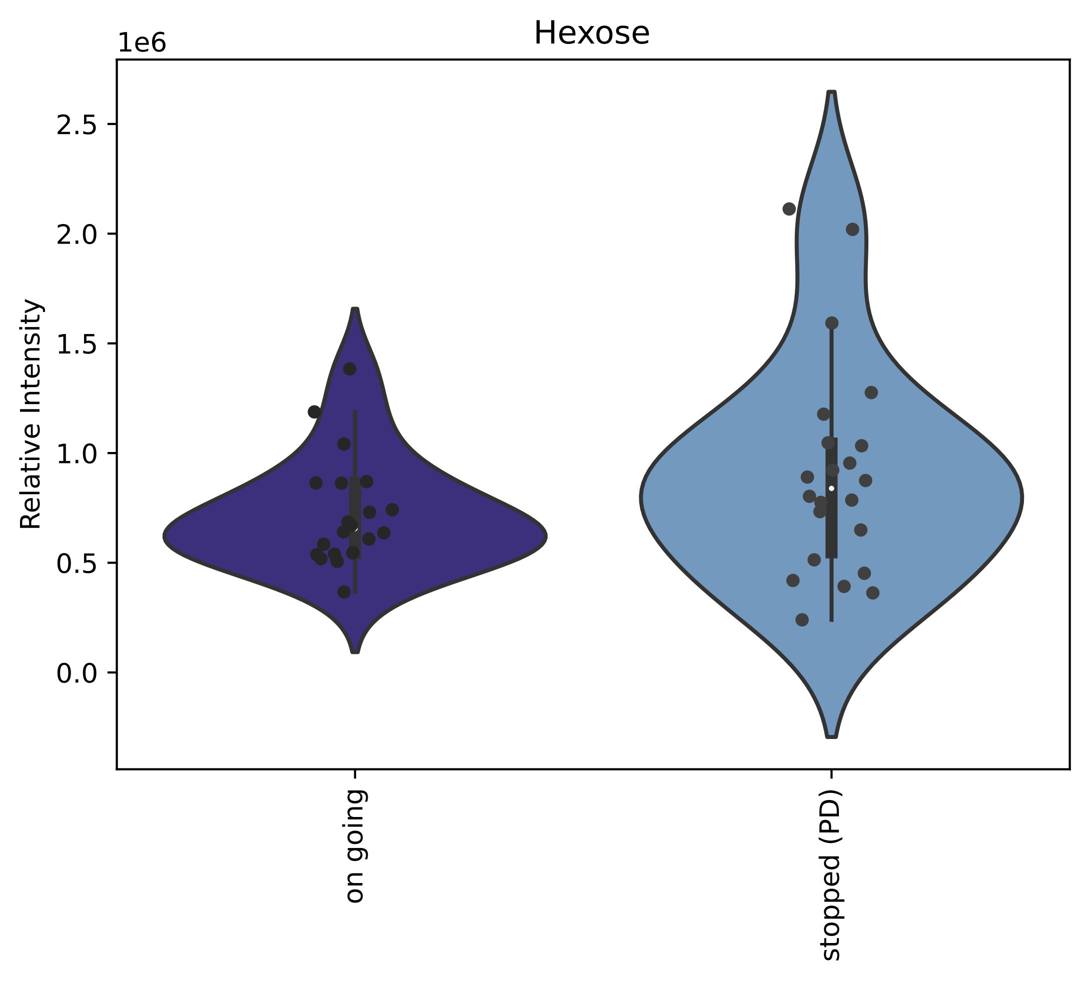

Hexose
1e6
2.5
2.0
1.5
1.0
0.5
0.0
Relative Intensity
on going
stopped (PD)

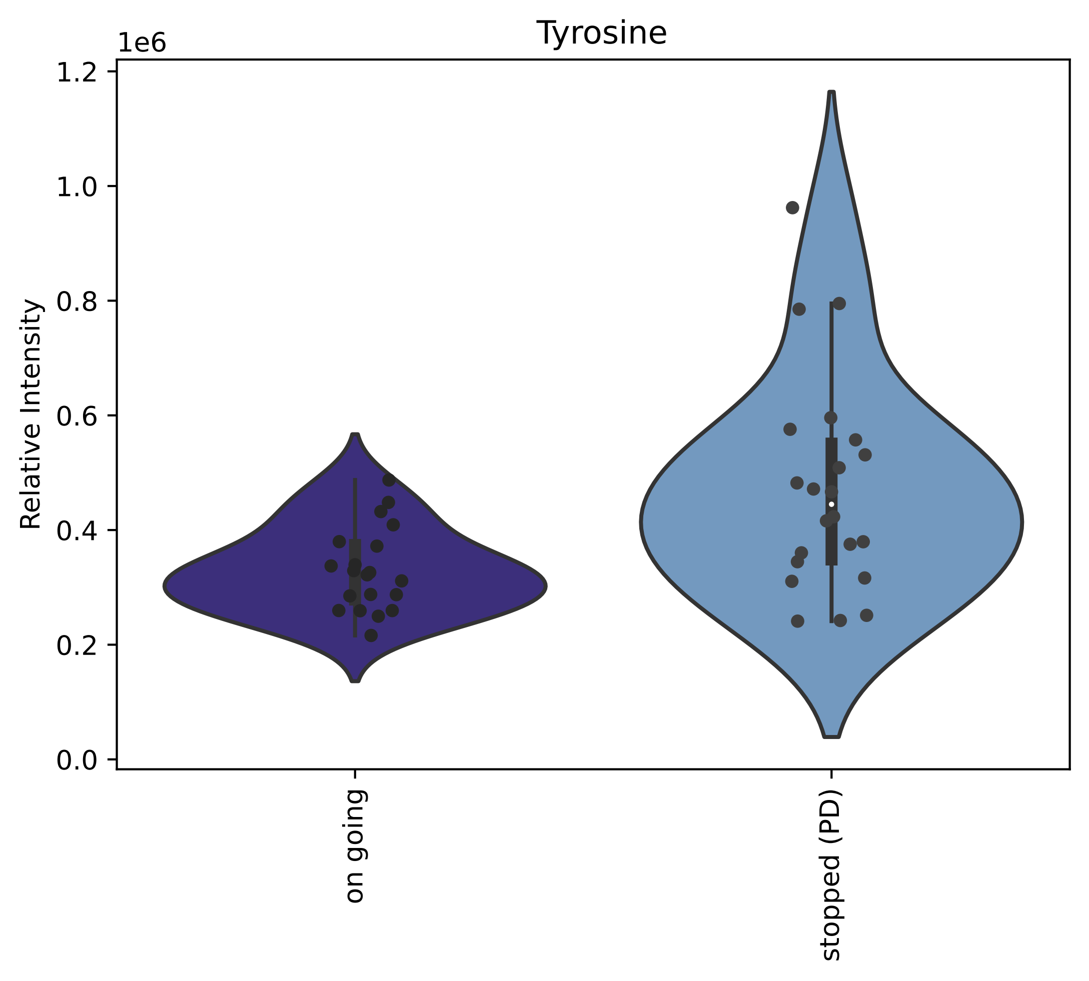
Tyrosine
1e6
1.2
1.0
0.8
0.6
0.4
0.2
0.0
Relative Intensity
on going
stopped (PD)

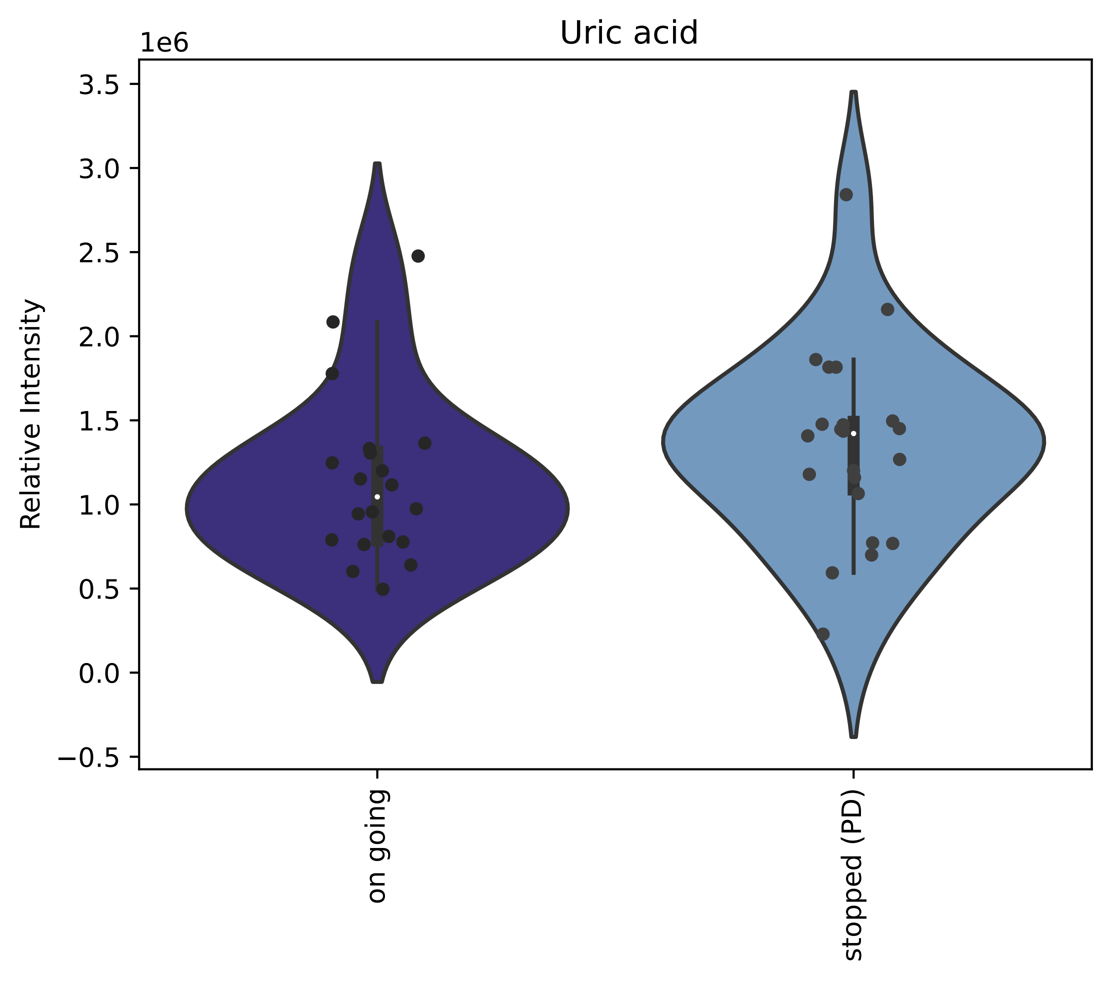
Uric acid
1e6
3.5
3.0
2.5
2.0
1.5
1.0
0.5
0.0
−0.5
Relative Intensity
on going
stopped (PD)

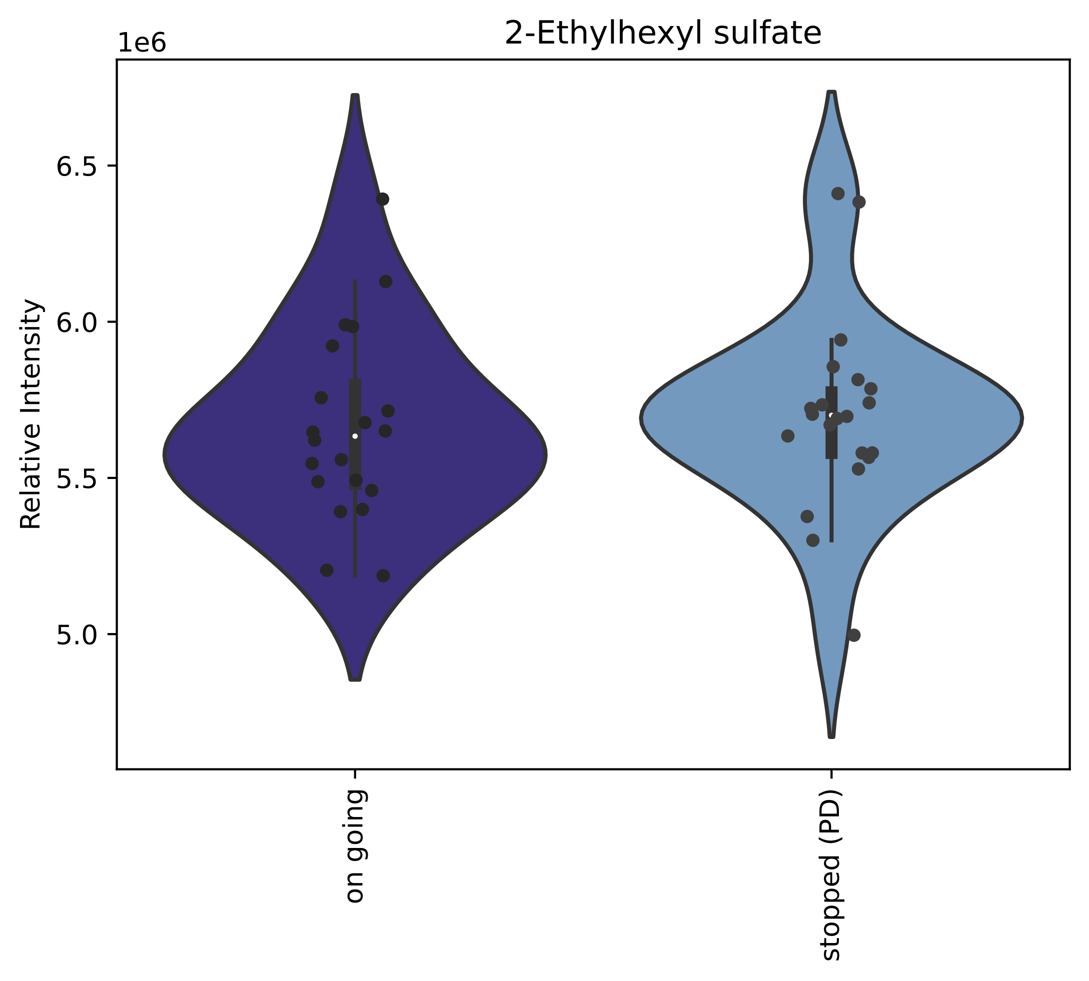

2-Ethylhexyl sulfate
1e6
6.5
6.0
5.5
5.0
Relative Intensity
on going
stopped (PD)

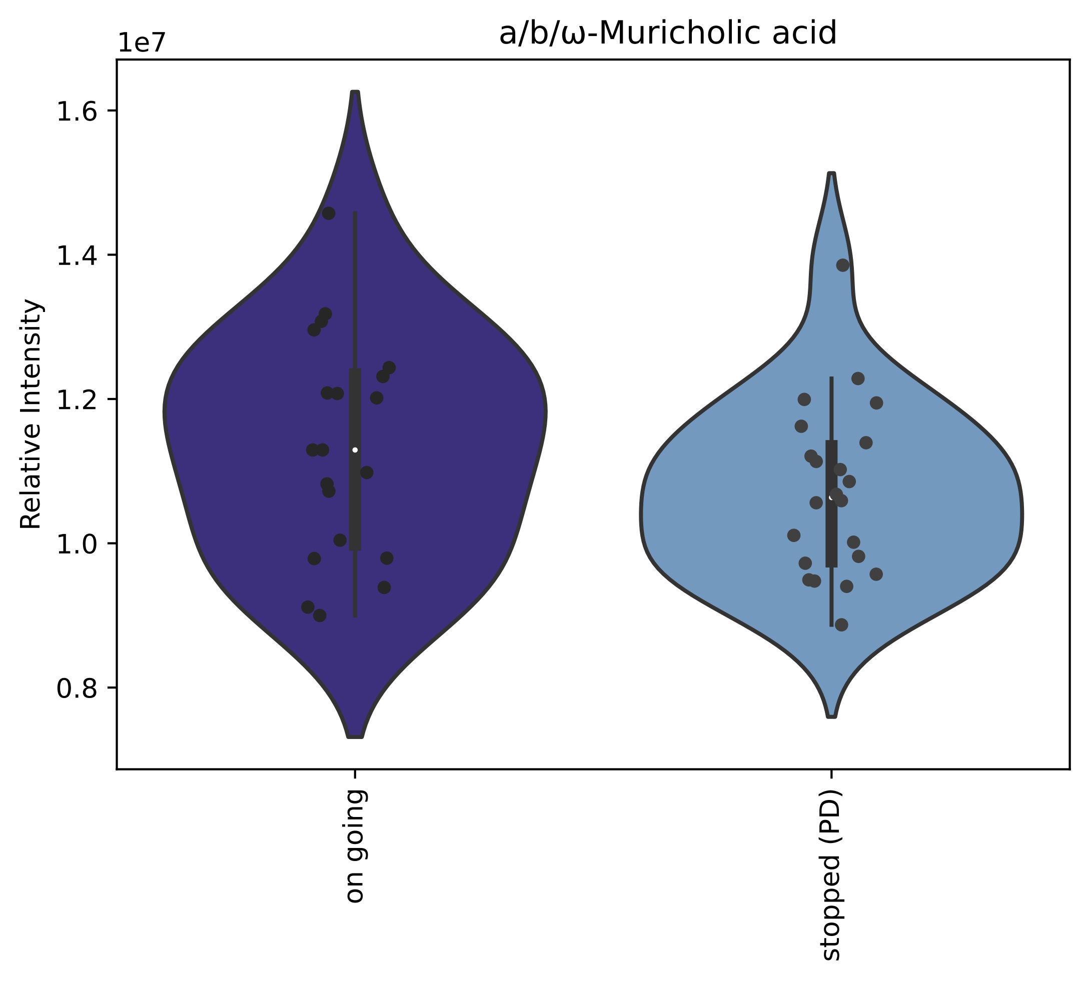

a/b/ω-Muricholic acid
1e7
1.6
1.4
1.2
1.0
0.8
Relative Intensity
on going
stopped (PD)

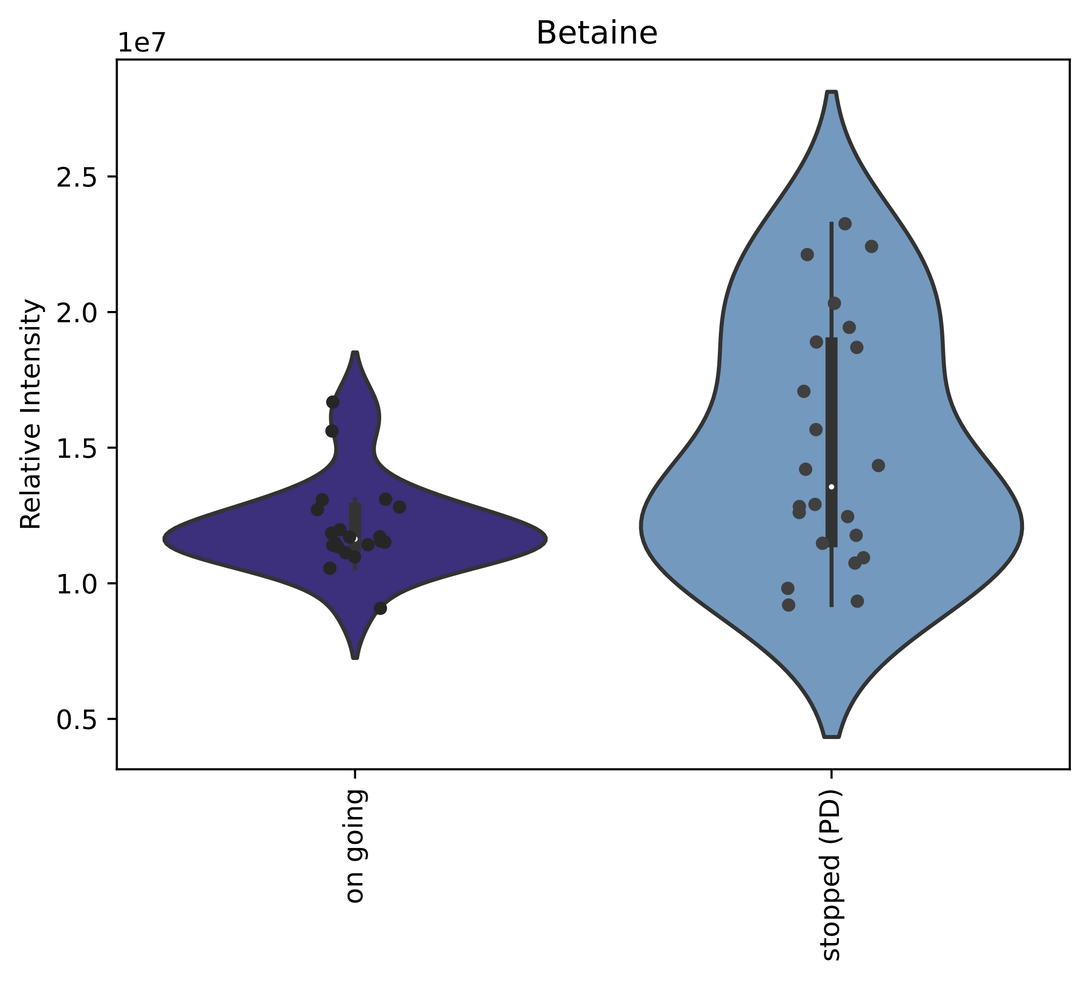

Betaine
1e7
2.5
2.0
1.5
1.0
0.5
Relative Intensity
on going
stopped (PD)

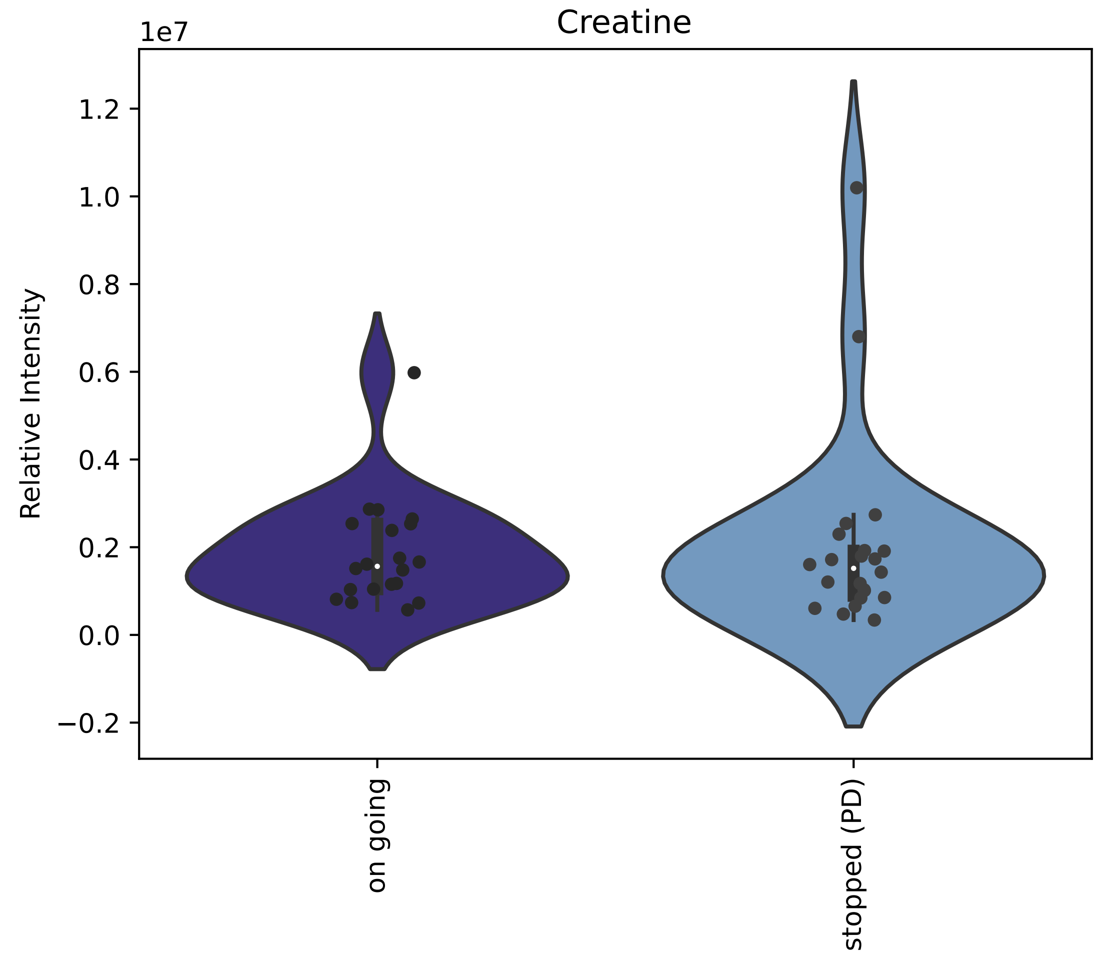

Creatine
1e7
Relative Intensity
1.2
1.0
0.8
0.6
0.4
0.2
0.0
−0.2
on going
stopped (PD)

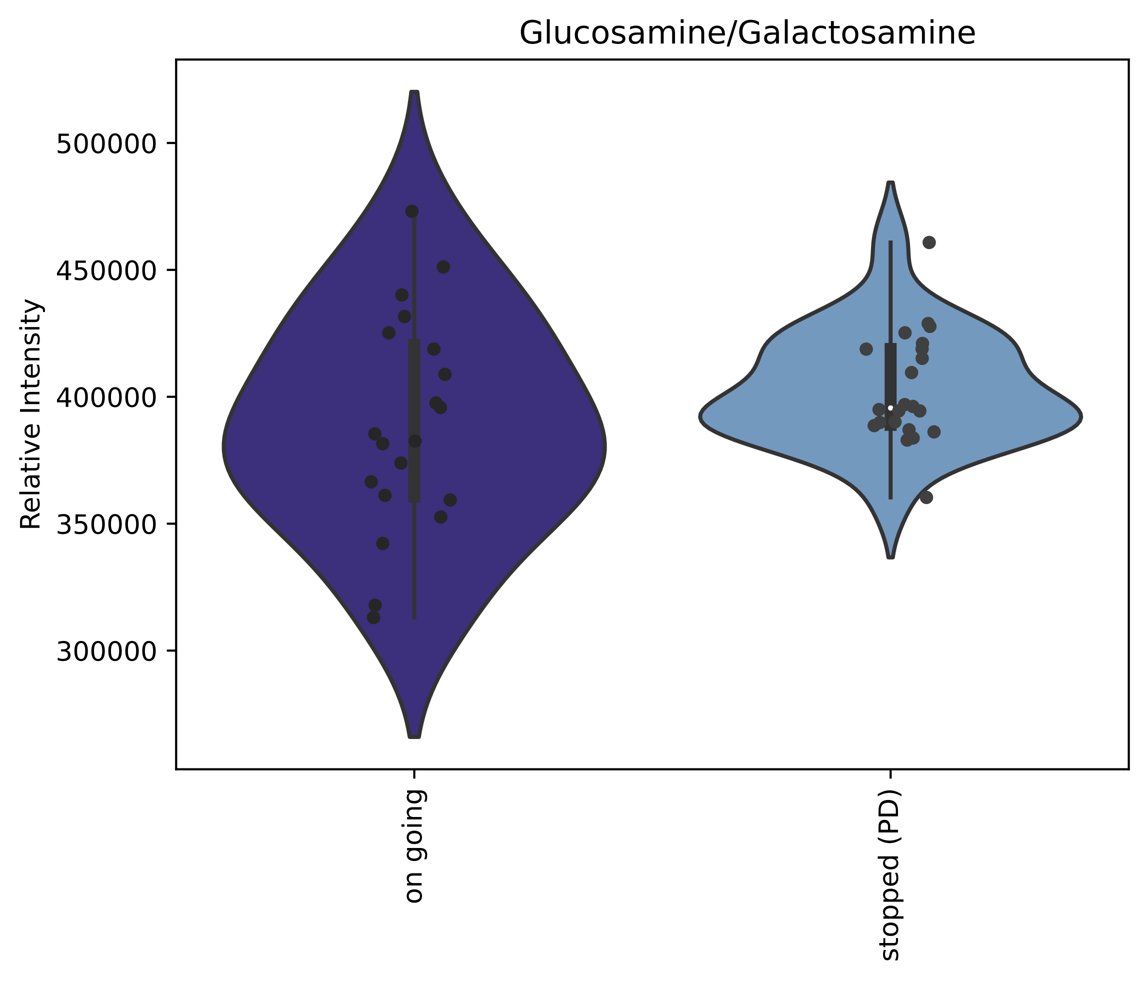
Glucosamine/Galactosamine
Relative Intensity
500000
450000
400000
350000
300000
on going
stopped (PD)

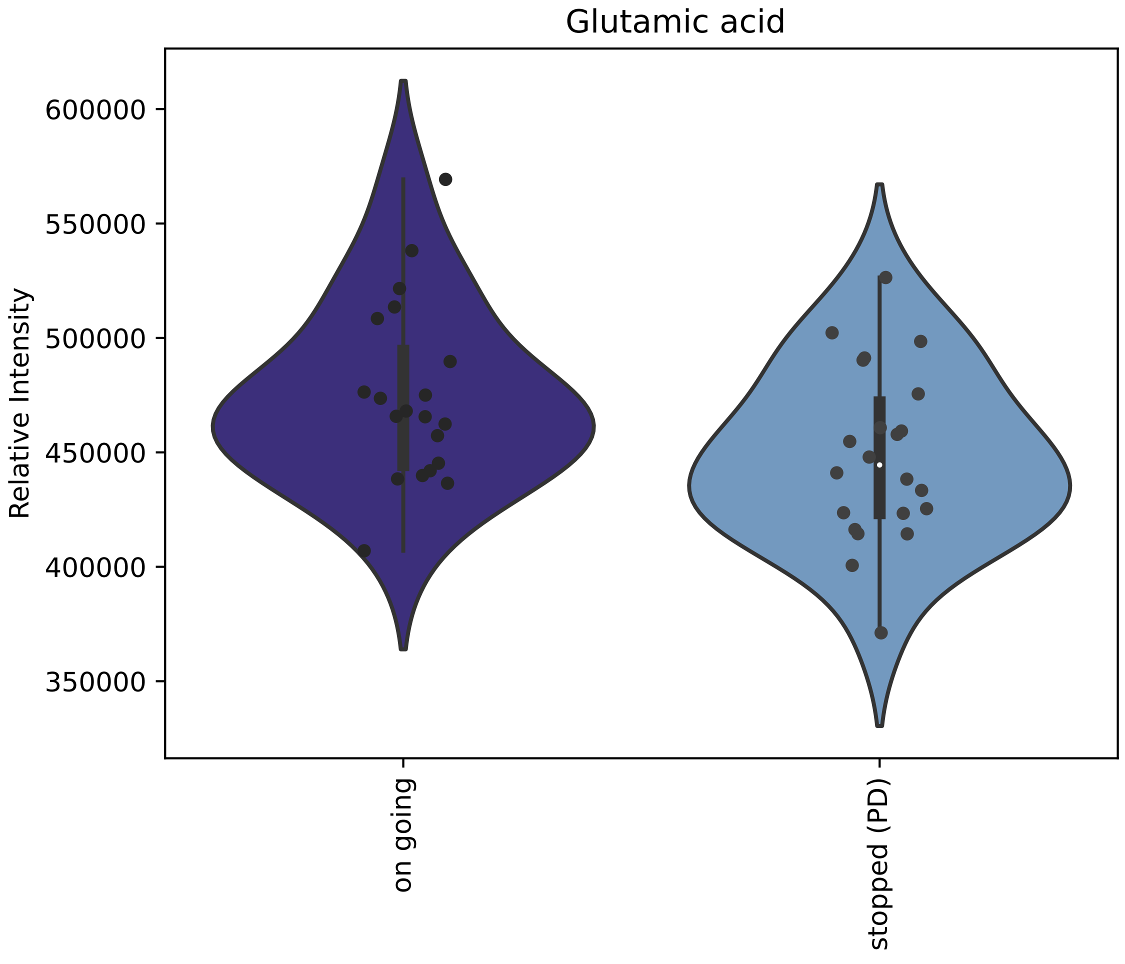

Glutamic acid
Relative Intensity
600000
550000
500000
450000
400000
350000
on going
stopped (PD)

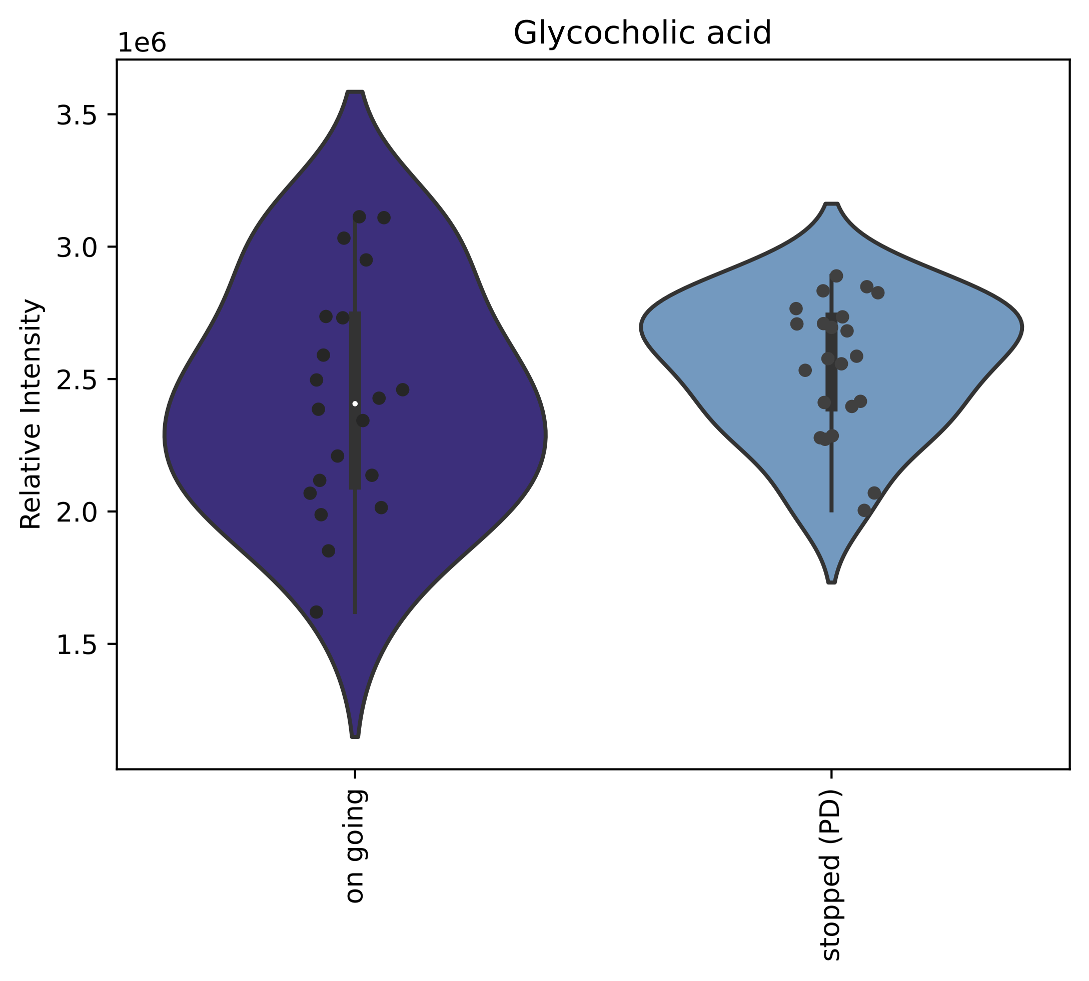

Glycocholic acid
1e6
3.5
3.0
2.5
2.0
1.5
Relative Intensity
on going
stopped (PD)

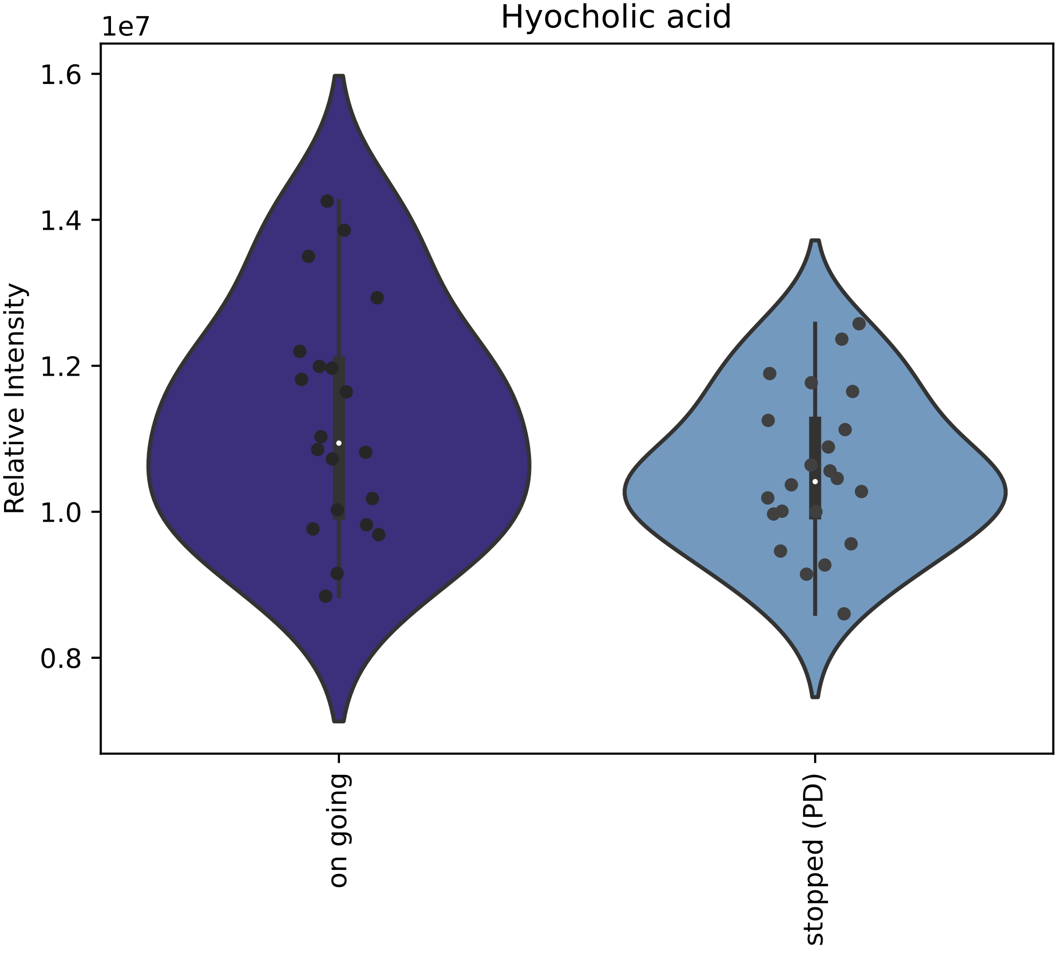

Hyocholic acid
1e7
1.6
1.4
1.2
1.0
0.8
Relative Intensity
on going
stopped (PD)

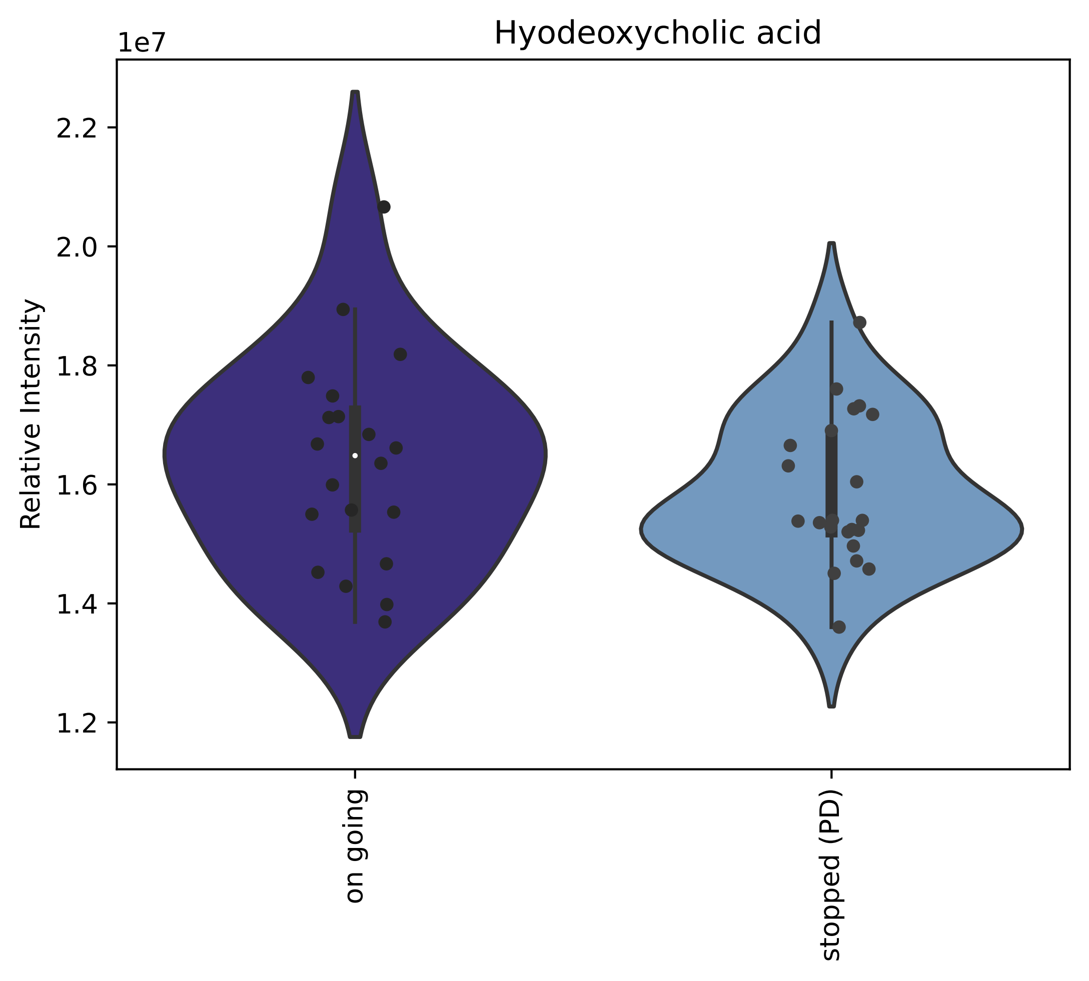
Hyodeoxycholic acid
1e7
2.2
2.0
1.8
1.6
1.4
1.2
Relative Intensity
on going
stopped (PD)

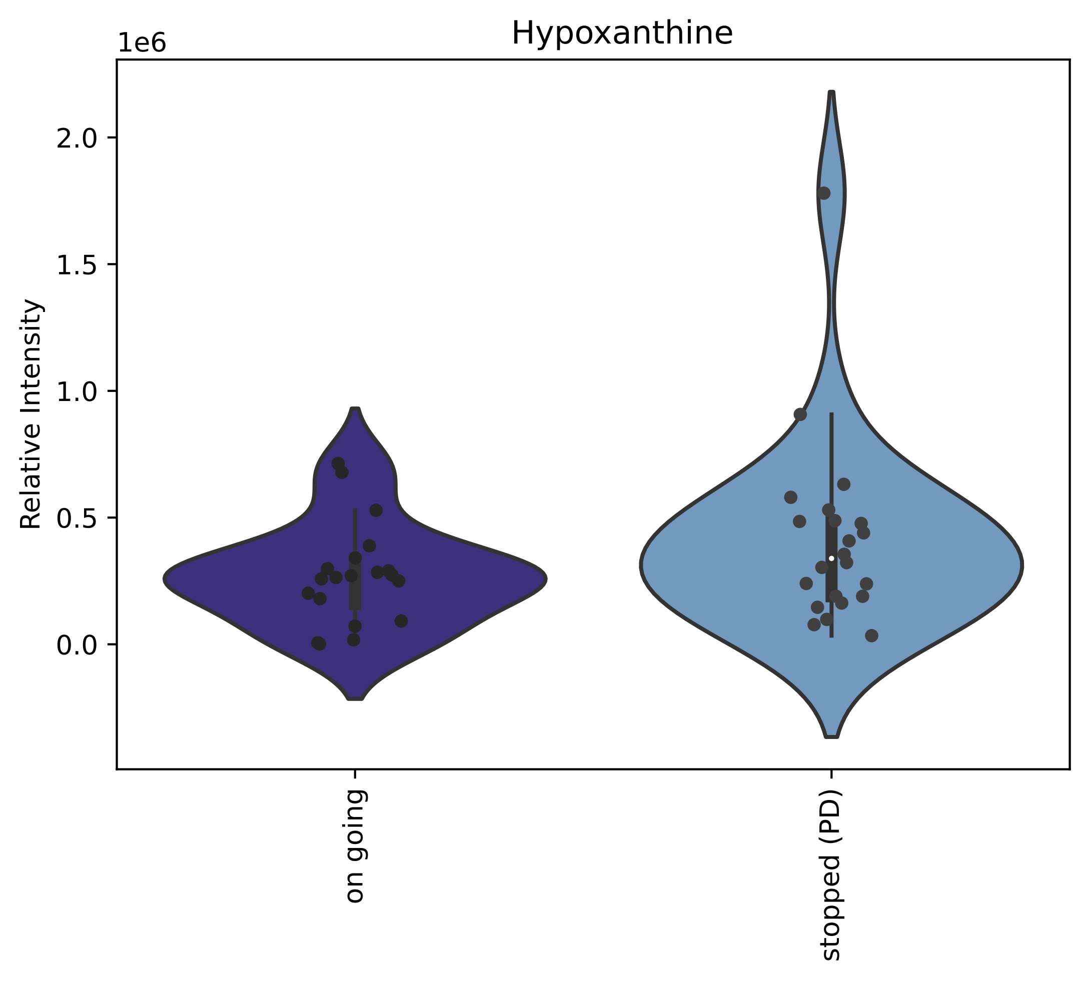

Hypoxanthine
1e6
2.0
1.5
1.0
0.5
0.0
Relative Intensity
on going
stopped (PD)

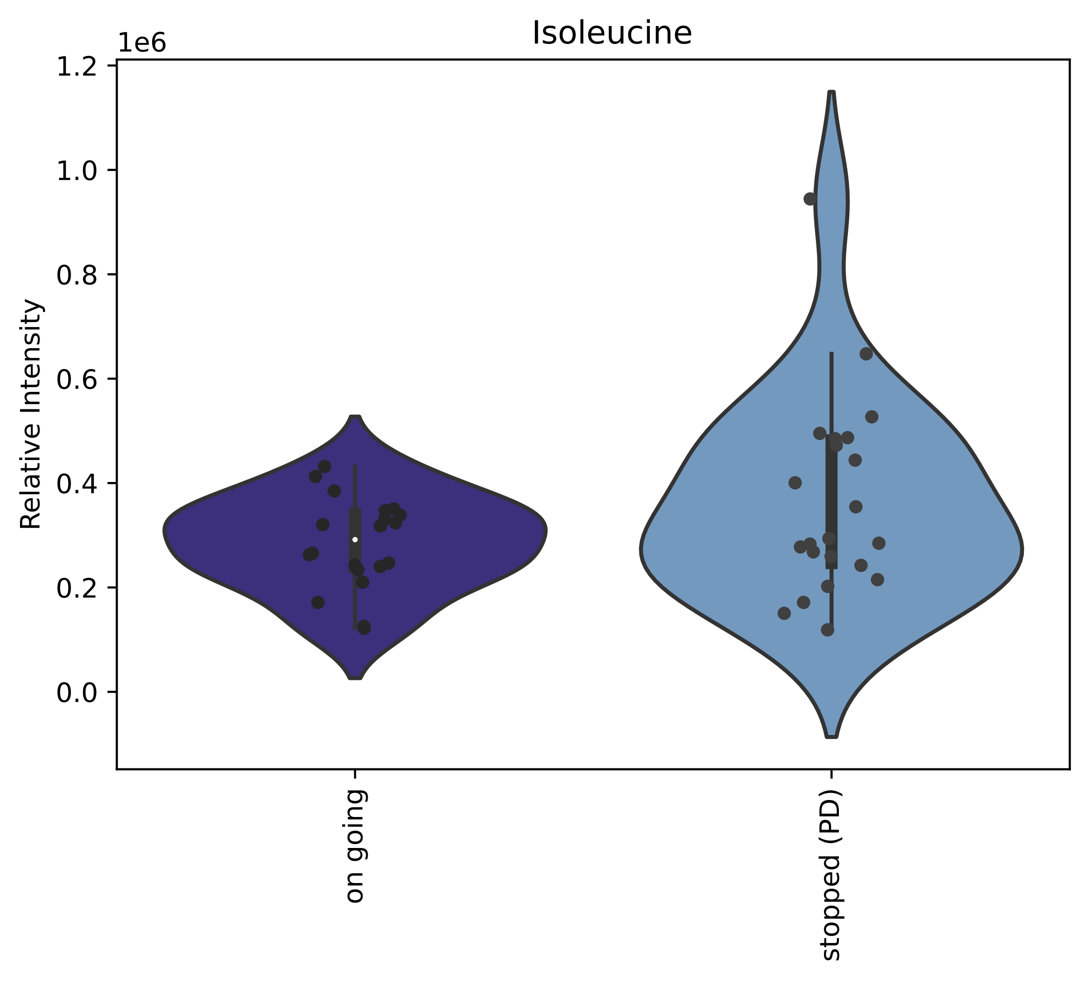

Isoleucine
1e6
1.2
1.0
0.8
0.6
0.4
0.2
0.0
Relative Intensity
on going
stopped (PD)

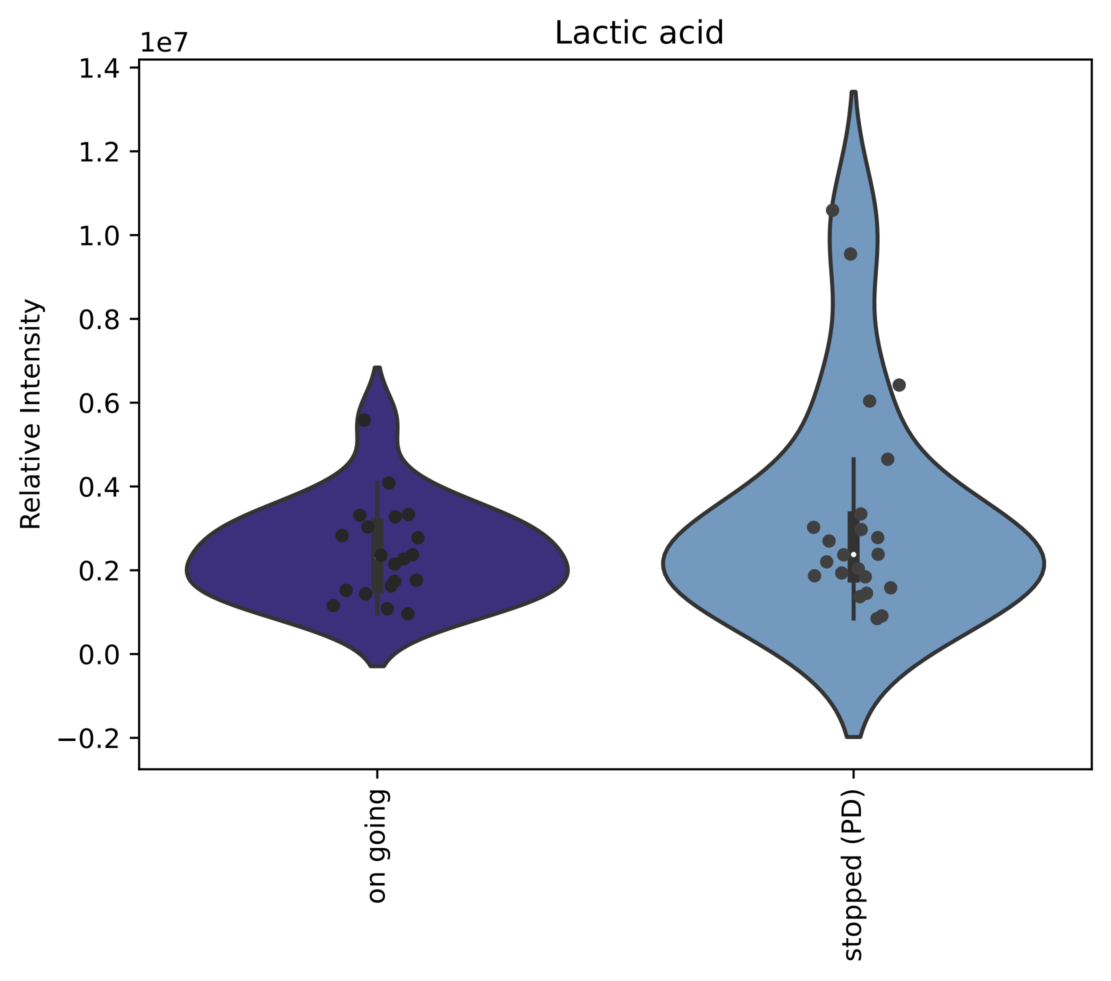
Lactic acid
1e7
1.4
1.2
1.0
0.8
0.6
0.4
0.2
0.0
−0.2
Relative Intensity
on going
stopped (PD)

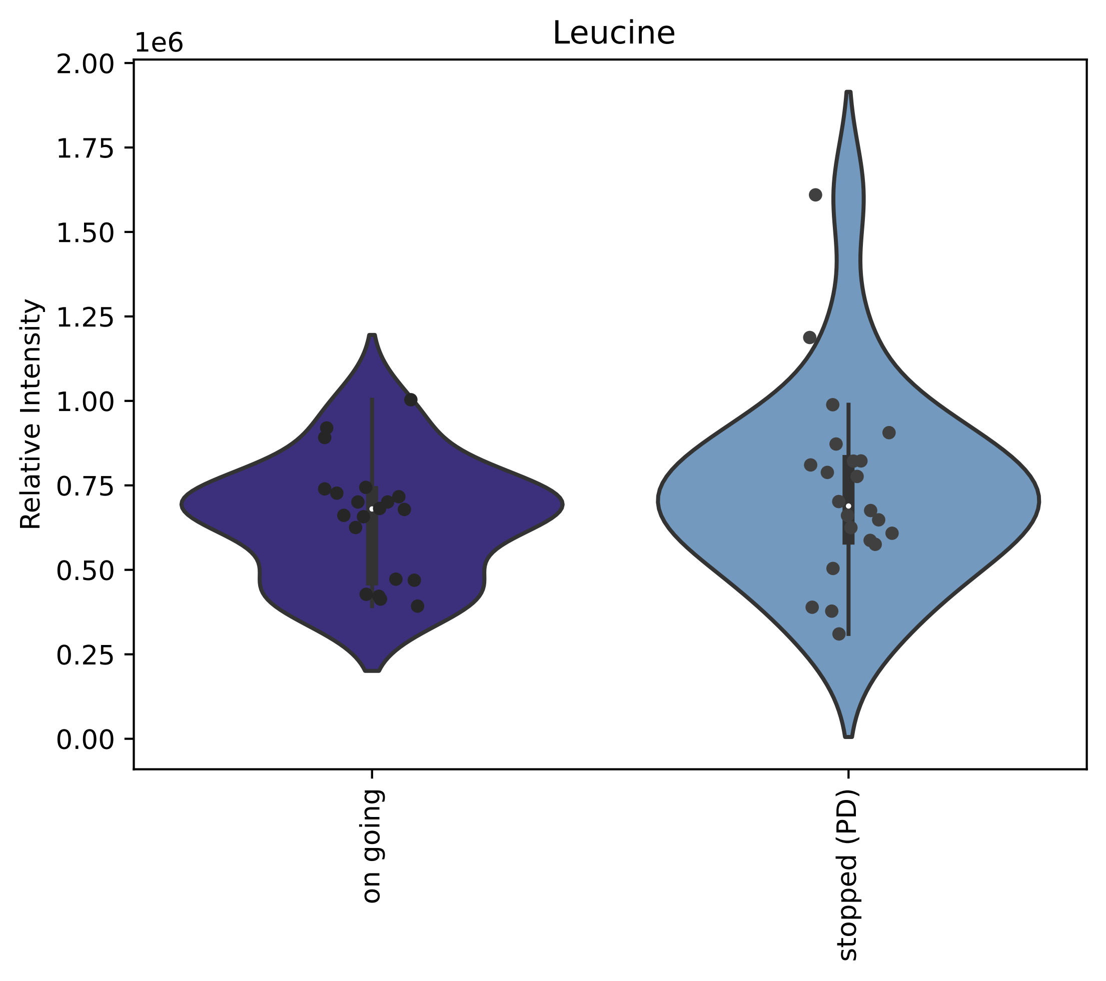

Leucine
1e6
2.00
1.75
1.50
1.25
1.00
0.75
0.50
0.25
0.00
Relative Intensity
on going
stopped (PD)

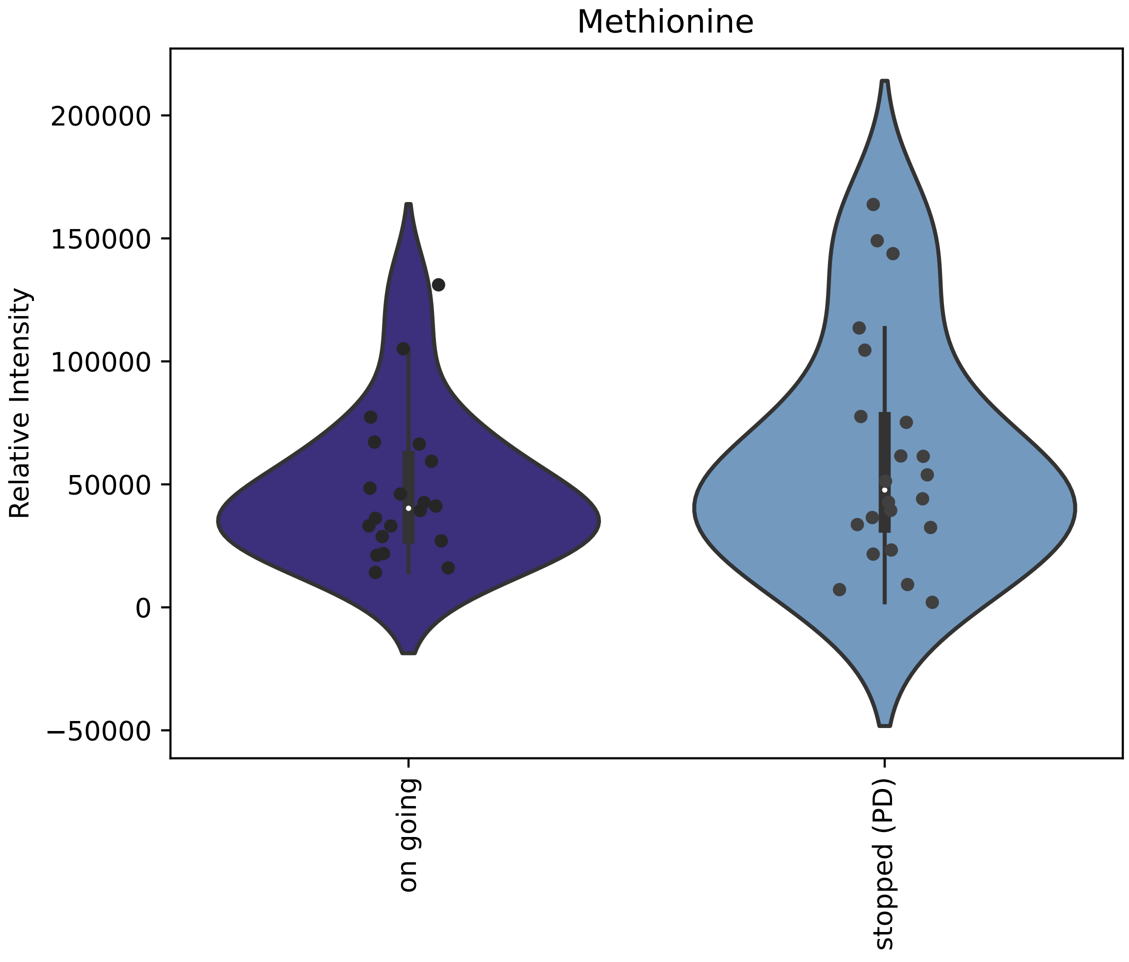
Methionine
Relative Intensity
200000
150000
100000
50000
0
−50000
on going
stopped (PD)

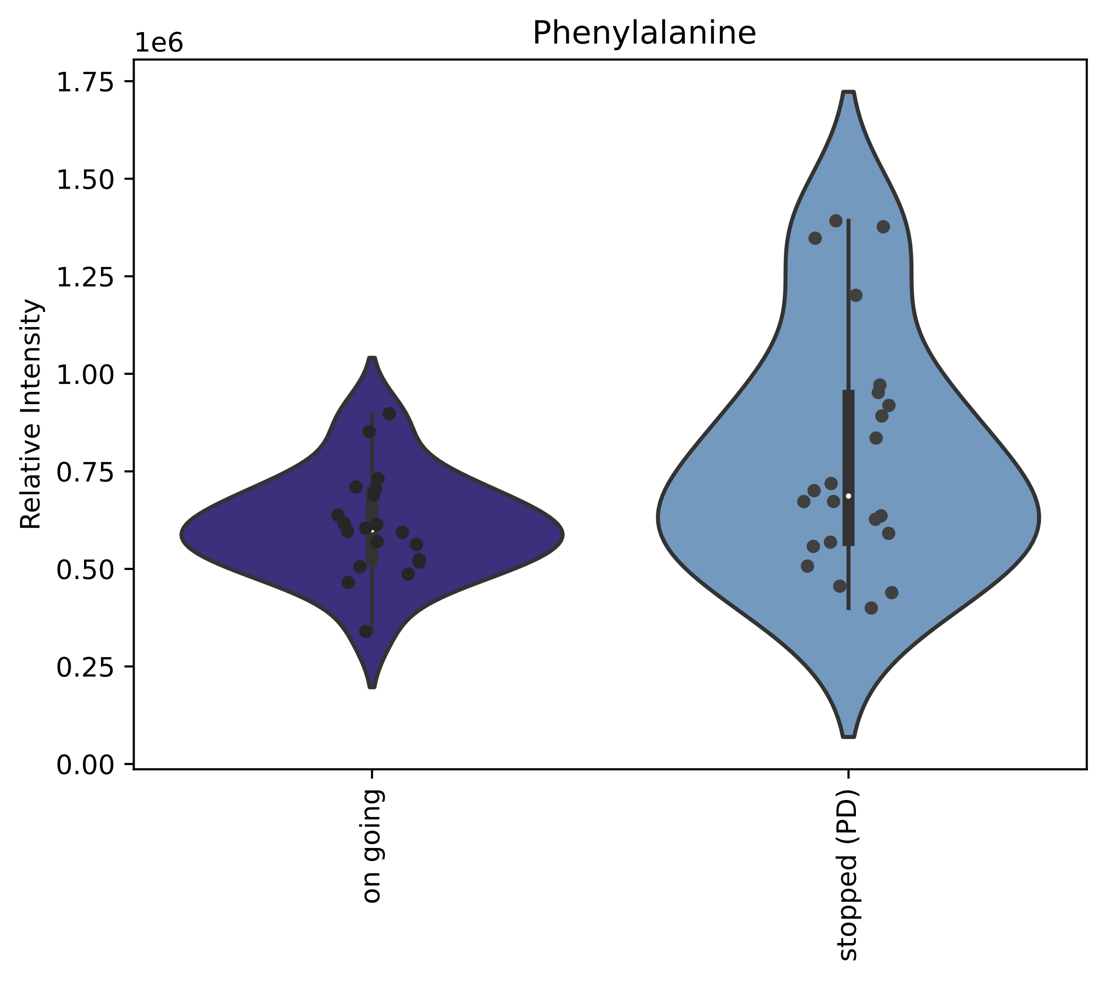
Phenylalanine
1e6
1.75
1.50
1.25
1.00
0.75
0.50
0.25
0.00
Relative Intensity
on going
stopped (PD)

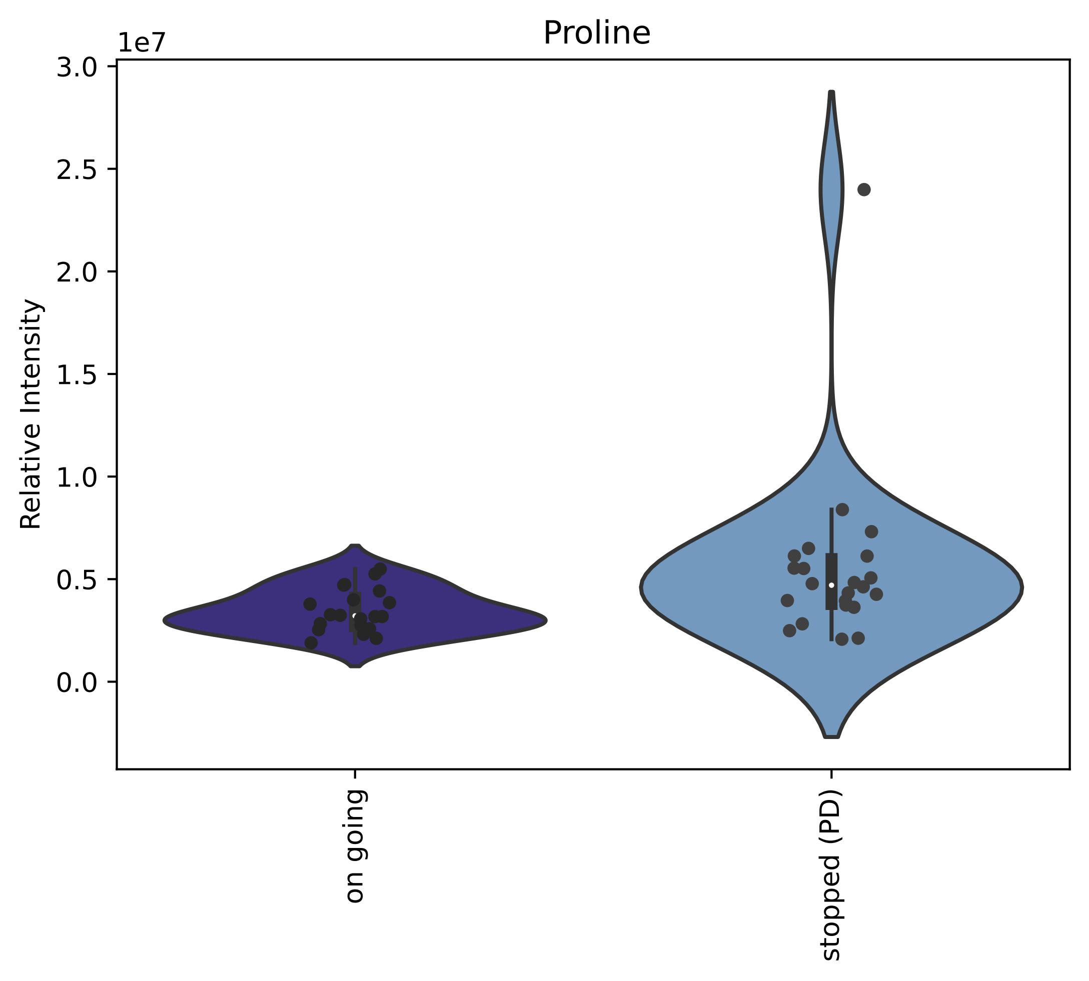
Proline
1e7
3.0
2.5
2.0
1.5
1.0
0.5
0.0
Relative Intensity
on going
stopped (PD)

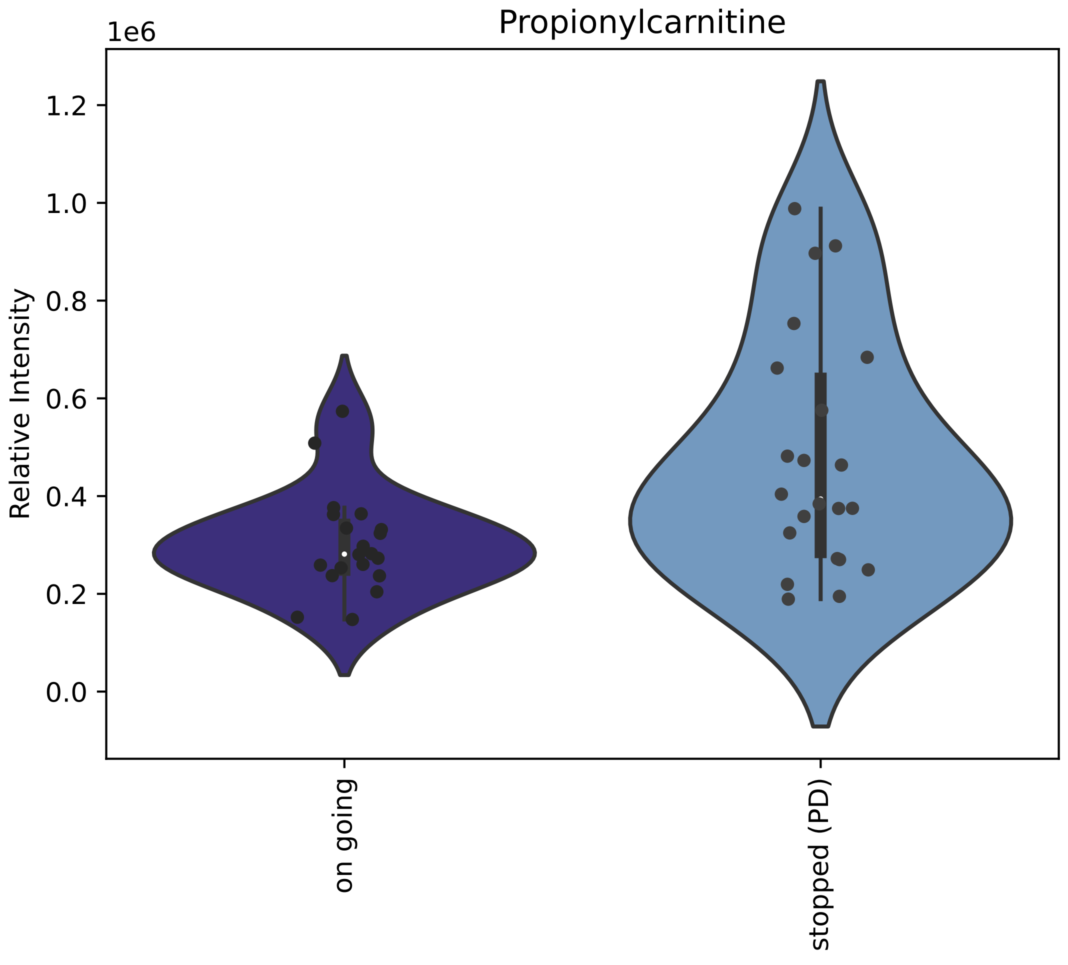

Propionylcarnitine
1e6
Relative Intensity
1.2
1.0
0.8
0.6
0.4
0.2
0.0
on going
stopped (PD)

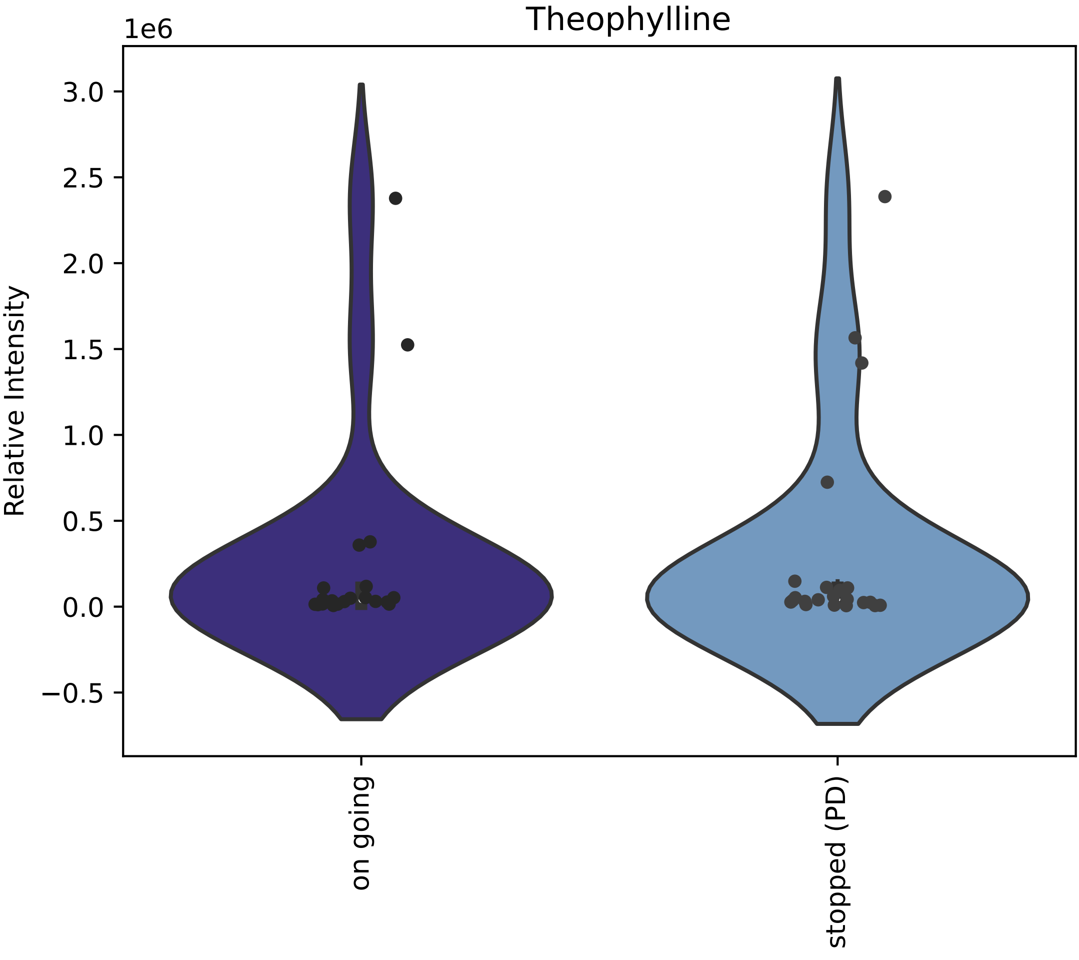

Theophylline
1e6
3.0
2.5
2.0
1.5
1.0
0.5
0.0
−0.5
Relative Intensity
on going
stopped (PD)

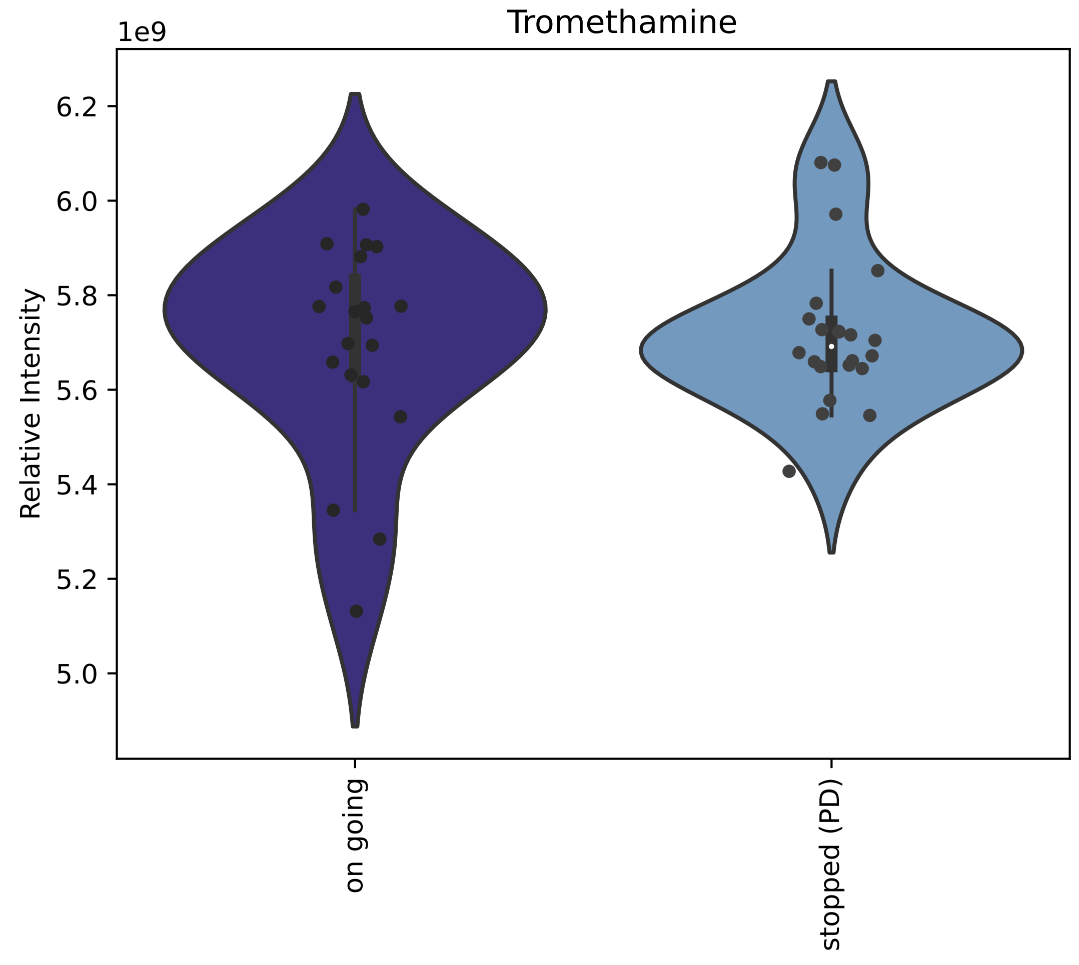

Tromethamine
1e9
6.2
6.0
5.8
5.6
5.4
5.2
5.0
Relative Intensity
on going
stopped (PD)

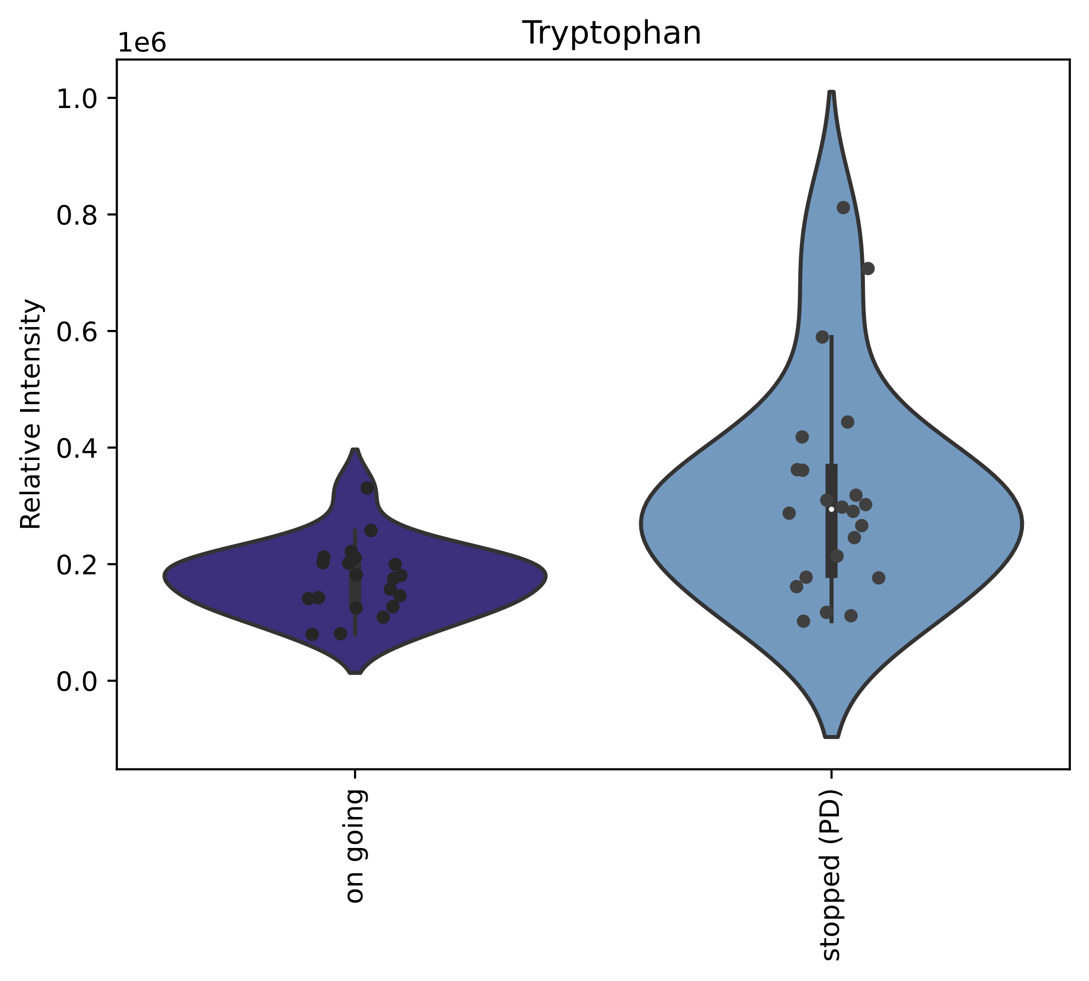
Tryptophan
1e6
1.0
0.8
0.6
0.4
0.2
0.0
Relative Intensity
on going
stopped (PD)

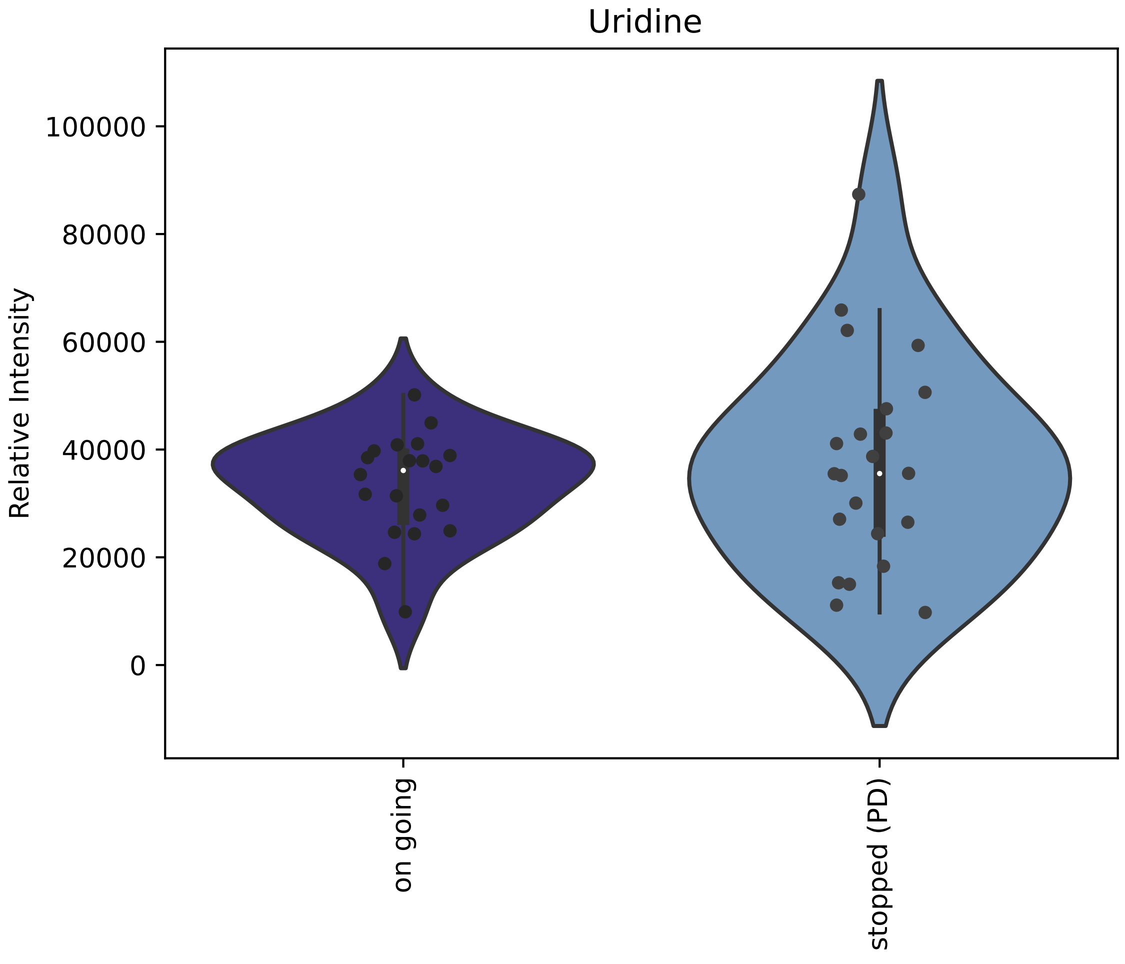
Uridine
Relative Intensity
100000
80000
60000
40000
20000
0
on going
stopped (PD)
